# Encountering shoaling internal waves on the dispersal pathway of the pearl river plume in summer

**DOI:** 10.1038/s41598-020-80215-2

**Published:** 2021-01-13

**Authors:** Jay Lee, James T. Liu, I-Huan Lee, Ke-Hsien Fu, Rick J. Yang, Wenping Gong, Jianping Gan

**Affiliations:** 1 Department of Oceanography, National Sun Yat-Sen University, Kaohsiung, Taiwan ROC; 2 Marine Science and Information Research Center, National Academy of Marine Research, Kaohsiung, Taiwan ROC; 3 School of Marine Sciences, Sun Yat-Sen University, Guangzhou, Guangdong China; 4 Department of Ocean Science & Department of Mathematics, School of Science, The Hong Kong University of Science and Technology, Hong Kong, China

**Keywords:** Hydrology, Ocean sciences

## Abstract

Fundamentally, river plume dynamics are controlled by the buoyancy due to river effluent and mixing induced by local forcing such as winds and tides. Rarely the influence of far-field internal waves on the river plume dynamics is documented. Our 5-day fix-point measurements and underway acoustic profiling identified hydrodynamic processes on the dispersal pathway of the Pearl River plume. The river plume dispersal was driven by the SW monsoon winds that induced the intrusion of cold water near the bottom. The river effluent occupied the surface water, creating strong stratification and showing on-offshore variability due to tidal fluctuations. However, intermittent disruptions weakened stratification due to wind mixing and perturbations by nonlinear internal waves (NIWs) from the northern South China Sea (NSCS). During events of NIW encounter, significant drawdowns of the river plume up to 20 m occurred. The EOF deciphers and ranks the contributions of abovementioned processes: (1) the stratification/mixing coupled by wind-driven plume water and NIWs disruptions (81.7%); (2) the variation caused by tidal modulation (6.9%); and (3) the cold water intrusion induced by summer monsoon winds (5.1%). Our findings further improve the understanding of the Pearl River plume dynamics influenced by the NIWs from the NSCS.

## Introduction

Significant physical and biogeochemical effects caused by internal waves (IWs) have been ubiquitously recognized^1–3^. A well-known example is that IWs play an important role in nutrient supply, which enhances phytoplankton primary productivity near the surface^4,5^. At the Mid-Atlantic Ridge, IWs redistributed nutrients to support phytoplankton growth^6,7^. In Southern California Bight, IWs strengthened the horizontal and vertical water displacement to develop a nutrient supply system when the water advection was weak^8,9^. When IWs propagate into the shallow coastal region, they evolve into various types such as the nonlinear internal waves (NIWs) along isopycnal surfaces^4,10–12^.

One of well-known examples is the case in the South China Sea (SCS). The NIWs are generated when the tidal flows over sills or ridges in Luzon Strait, and then propagating westward as the mode-1 internal tides^13–15^. The shoaling bottom topography along the wave propagation pathway induces the polarity reversal of NIWs which turns from the depression wave to the elevation wave^10,13^. The reasons causing the turning point of the polarity reversal include the depth of the mixed layer in the water column or the ratio of upper and lower layers. Both depression wave and elevation wave could occur in the same areas even though within different seasons^14^.

The NIW also contributes to bottom-boundary turbulence when it is propagating downstream and induces sediment resuspension in the SCS, resulting in the formation of benthic and intermediate nepheloid layers^1,16^. As the NIWs pass, strong water movements enhance the dilution rate of water-borne substances and facilitate the water exchange in coastal and open oceans, altering the seawater stratification temporally^14,17,18^. However, there is a lack of knowledge regarding the influence of the NIWs on the shelf-mixing processes and coastal river plume hydrodynamics which is an important factor to influence the water stratification in nearshore area.

Conceptually, mixing processes in coastal areas are mainly affected by the strength of stratification as well as the strength of forcing-induced shear^19,20^. The water-column stability is reinforced when hypopycnal river plume enters the ambient coastal water^21–25^. By contrast, mixing induced by both the *in-situ* and distal forcing influences the stratification and causes the intrinsic water column properties (e.g. salinity and temperature etc.) to fluctuate temporally and spatially^21,26,27^. For example, the current-induced mixing modified the sea surface temperature due to the nonlinear interaction between diurnal and semi-diurnal tides^28^. The typhoons affected the local wind field in direction and strength, creating a well-developed mid-depth pycnocline layer in the water column^21,29^. The plume-related NIWs occurring at plume front displaced the surface water mass downward within a very short period and enhanced the mixings between river plume and adjacent sea^30,31^. These processes indicate complex interactions exist among river plume and the ambient coastal water through river plume dynamics (buoyancy), local forcing (wind and current mixing), and far-field forcing (typhoons).

The dispersal of the river plume in the coastal region is predominantly affected by the hydrodynamic processes^32,33^. Chao and Boicourt^34^ indicated that the wind-generated across-shore current constrained the river plume to disperse seaward. Liu et al.^35^ showed the dispersal of river plume was jointly controlled by the tidal current and the wind by using POM (Princeton Ocean Model). In order to clarify the dispersal processes of the riverine substances from their terrestrial sources to coastal sinks, it merits further investigation in the case of a major river such as the Pearl River, which is considered the major source of nutrients (e.g. NO_3_ etc.) to the Taiwan Strait in summer and, in turn, increases the primary production along the path of the plume^24,36–40^.

The discharge of the Pear River could be as large as ~ 2.1 × 10^4^ m^3^/s in the wet season^41^. After exiting the funnel-shaped Pearl River Estuary (PRE), the terrestrial effluent affected the characteristics and biogeochemistry of water masses along the pathway of the plume^36^. The northeastward dispersal of the Pear River plume largely depends on the wind field and coastal currents^37,42,43^. Under the prevailing upwelling-favorable monsoon winds, the buoyant river plume is trapped and propagating northeastward further along the upwelling front because of the strong cross-shore temperature gradient. The upwelling, based on a previous modelling study^38^, might trigger the intrusion of low temperature offshore water near the bottom in the cross-shore direction off Guangdong coast^37,44^. The northwest-southeast oriented tidal currents produced additional complexity to the upwelling circulation, of which the influence was not significant because of the small tidal range (< 2 m), indicating a micro-tidal environment^41,45^.

Although the plume dynamics and circulation systems in this region have been well documented^37,38,41,43^, most studies are based on model simulations as well as spatial observation along transects. This might cause the time and space aliasing issues from the observations and, consequently, lose the temporal variability of the water-mass signatures under the plume-ambient water interaction on the river plume pathway.

**Figure 1:**
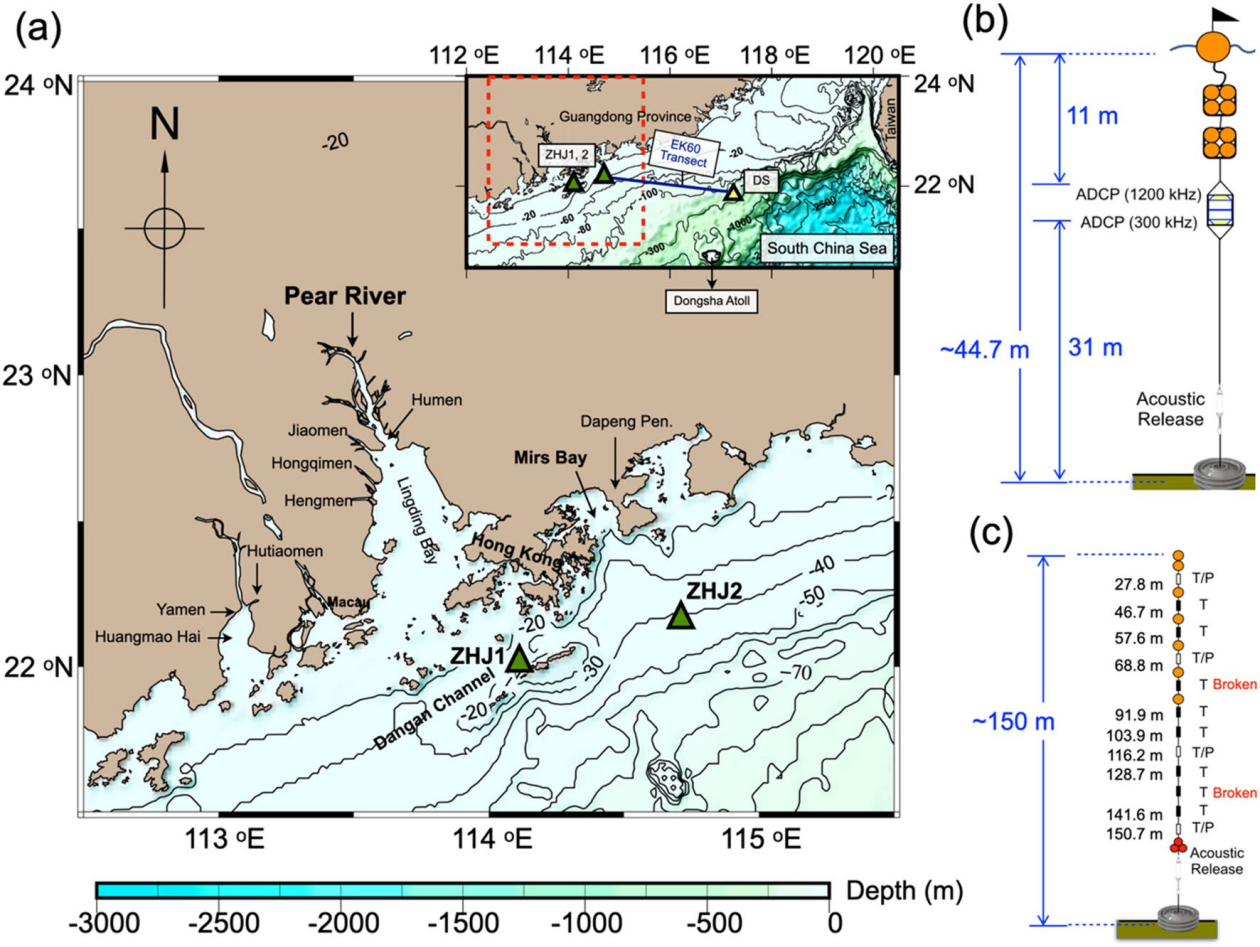
A map showing the area of interest in northern South China Sea with configuration of deployed moorings. (**a**) The map (plotted using the Generic Mapping Tools (GMT, V5.4.2, https://www.soest.hawaii.edu/gmt/) shows the locations the two monitoring sites (green triangles) in June (ZHJ1) and July (ZHJ2) of 2016. The insert is a larger-scaled map showing the location of the Dongsha Sta. (DS, yellow triangle) on the northern edge of the South China Sea basin where temperature mooing was deployed. The echogram transect recorded by EK60 is depicted by the blue line. (**b**, **c**) are the configuration of moorings deployed in ZHJ2 and the SCS (yellow triangle). The symbol T and T/P in (c) indicates the 8-bit mini-logs and DST milli-TD, respectively.

Therefore, this study designed a comprehensive plan to observe temporal variability of water-column structures at two fixed locations in June and July 2016. The location in June (ZHJ1 for 42 h) was in the vicinity of the opening of the PRE and the other in July (ZHJ2 for 120 h) was on the plume path, 98 km away from the mouth of PRE (Fig. 1a). The wind field at both sites were recorded by the meteorological stations onboard the research vessels (Supplementary Figs. 1a, 2a). Shipboard ADCPs and the current profilers on the mooring nearby were used to measure the flow field (Fig. 1b). Only the hourly hydrographic profiling data obtained at ZHJ2 are presented in this paper because of technical issues at ZHJ1. In addition, the acoustic backscatter strength measured by the high-resolution echo sounder (EK60) was used to identify the structure of NIWs in the water column (EK60 transect; Fig. 1a). Another mooring mounted with 12 mini-logs was deployed to record the water temperature at different depths from June 2nd to July 23th, 2016 north of the Dongsha Atoll (Sta. DS; Fig. 1a,c).

## Results

### Meteorological variability

Winds measured at both sites were mostly southerly/southwesterly (Supplementary Figs. 1b, 2a), which were occasionally interrupted by northerly/easterly winds coinciding with lower atmospheric pressure. At ZHJ2, a stormy weather event occurred between 18:00 July 25 and 11:00 July 27 in which the gust wind speed reached 13.7 m/s. This suggests the effect of the peripheral circulation of Tropical Storm Mirinae or the passing tropical rain belt^46^. The average wind speed was 4.3 m/s during the experiment at ZHJ2.

### Spatial variability of the coastal currents

The progressive vector (PV) plots of instantaneous currents at both sites show the net NE displacements (Supplementary Fig. 1c, 2b,c), which are the typical summer current patterns seaward of the PRE^37,41,45^. At ZHJ1, the non-tidal current (at 16.6 m) was largely flowing north-northeastward, along the Dangan Channel (Supplementary Fig. 1). The form number (F) indicates the tidal current was mainly composed of the semi-diurnal tide (Table 1). Superimposed by the M_2_ and K_1_ tidal components on the non-tidal flow (Supplementary Fig. 1c,d and Table. 1), the instantaneous current was in a zigzag pattern in northwest-southeast directions (on-offshore). However, the tidal energy ratio (ER) was 38.9%, suggesting that the tide was not the primary forcing to drive the instantaneous flow for the short observational period (Table 1). Near the end of the observation, the tidal signal in the instantaneous current became weak, and the current direction remained consistently northeastward. The average speed of the instantaneous current in the measurement was 0.18 m/s.

**Table 1:** The tidal constituents at different depths in in both cruises (June and July) and at DS site. Only the first four tidal components (K_1_, O_1_, M_2_, and S_2_) are listed in the table.

Station	Dep. (m)	K_1_ (m/s)		O_1_ (m/s)		M_2_ (m/s)		S_2_ (m/s)		ER (%)	F
		Major	Minor	Major	Minor	Major	Minor	Major	Minor		
June (ZHJ1)	16.6	0.06	0.00	x		0.18	0.01	x		38.9	0.19
July (ZHJ2)	1	0.16	0.09	x		0.11	0.01	x		17.3	1.7
	7	0.19	0.13			0.11	0.01			20.4	2.1
	14	0.02	0			0.07	0.01			12.0	0.3
	21	0.07	0.06			0.09	0.01			36.7	1.0
	28	0.07	0.06			0.09	0.01			49.3	1.0
	34	0.05	0.04			0.08	0			44.3	0.8

DS site	Dep. (m)	K_1_ (°C)		O_1_ (°C)		M_2_ (°C)		S_2_ (°C)		EsR (%)	F
		Major	Minor	Major	Minor	Major	Minor	Major	Minor		
	35	0.90	x	0.60	x	0.26	x	0.27	x	39.6	2.8
	55	1.33		1.00		0.33		0.39		49.0	3.2
	75	1.45		1.09		0.35		0.36		51.4	3.6
	95	1.49		1.11		0.29		0.24		50.0	4.9
	115	1.32		0.98		0.14		0.10		53.4	9.6
	135	0.97		0.70		0.12		0.05		55.8	9.8

The "x" symbol indicates the amplitude was insignificant or there was lack of data. The ER ratios indicates the tidal-to-total energy ratio, and F means form number.

**Figure 2:**
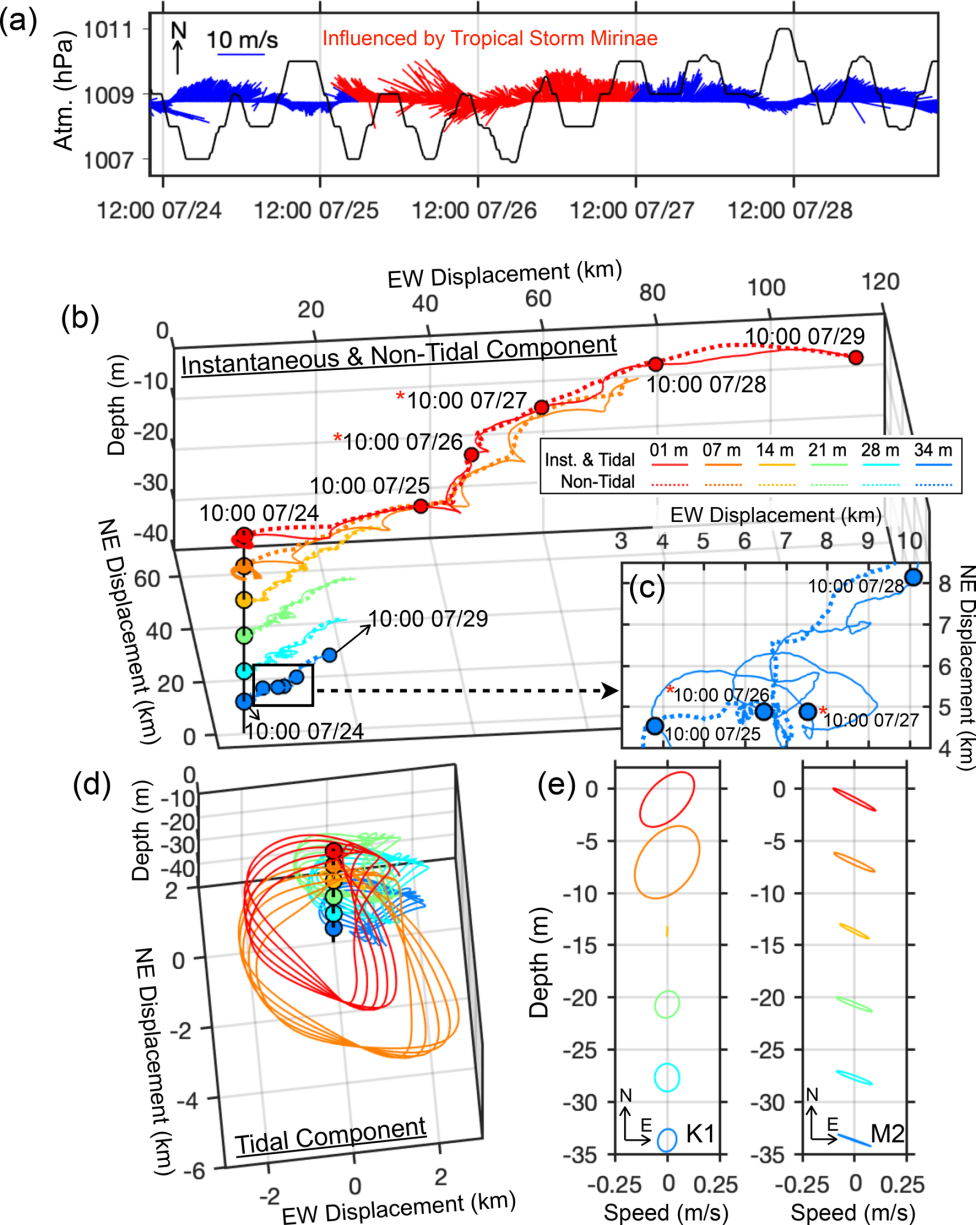
The meteorological background and current field measured at ZHJ2. (**a**) The stick diagram of the wind field and atmospheric pressure measured aboard the research vessels. The stick diagrams are plotted according to the oceanographic convention. The north is the upward direction. Both the wind and pressure fields were plotted at 20-min interval. The duration influenced by Tropical Storm Mirinae is marked by red stick. (**b**) The PV of the flow field (color-coded for different depths). Warm colors represent layers near the surface. Cold colors represent layers near the bottom. The instantaneous flow and non-tidal components are plotted as solid lines and dash lines, respectively. The times influenced by Tropical Storm Mirinae are marked with the red stars. (**c**) The enlarged portion of the PV at 34 m marked with the black square in (**b**). (**d**) and (**e**) are the 3-D view of tidal current component and tidal current ellipses at different depths with the same color codes.

At ZHJ2, the 5-day current observation showed the PV displacements of instantaneous currents rapidly decreased with depth, indicating the slower current speed in the lower water column (Fig. 2b and Supplementary Fig. 2). The average instantaneous current speed was 0.39 m/s at the surface, but only 0.12 m/s near the bottom. The differences between the surface and bottom flows were found not only in the current speed, but also in the current direction. Because of the interaction among the large-scale current^47^, bottom frictional effects^48^, and wind-induced Ekman pumping^47^, the current structure in the vertical mainly turned counterclockwise as the water depth increased (Fig. 2b and Supplementary Fig. 3). The instantaneous flow to ENE in the upper water column, and then the flow veered toward NE near the bottom layer. Additionally, the non-tidal bottom current showed stagnation between 10:00 July 26th and 10:00 July 27th (marked with a red star in Fig. 2b,c) though the surface current flowed consistently northeastward.

In general, the instantaneous flow at each depth was composed of a northeastward non-tidal component superimposed by a short circular tidal component (Fig. 2b,d). The tidal flow was basically composed of NE-SW orientated diurnal and NW–SE semi-diurnal tidal components (Fig. 2e). The F value indicates the tidal current was mixed with diurnal dominance in the upper water column and mixed with semi-diurnal dominance in the lower water column (Table 1). Overall, the K_1_ constituent was dominant at surface, and M_2_ became more important in the lower water column where the ER was over 40%. However, the tidal current at sub-surface layers (7 m) showed a significant increase of K_1_ strength (Fig. 2e and Table 1). This variability also showed in the F and ER value as a ‘sandwich structure’ above 21 m, in which an abrupt increase at 7 m and decrease at 14 m. This might be due to the NIWs perturbation in the current field.

**Figure 3:**
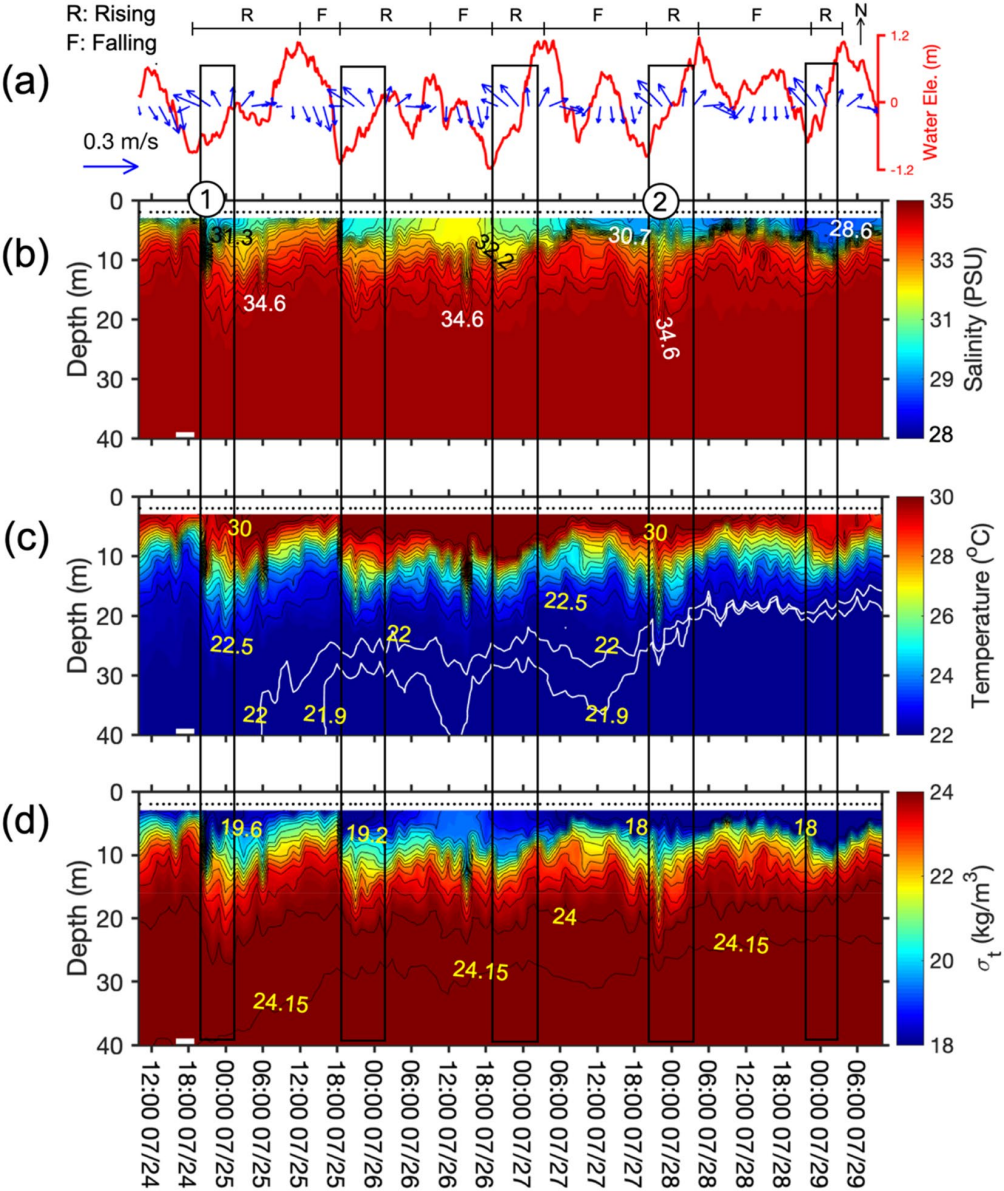
ADCP and shipboard measurements at ZHJ2. (**a**) stick diagram of mean tidal current averaged over the top 5 m superimposed with the sea-surface fluctuations (red line) recorded by ADCP. Text "R" and "F" above the sea-surface fluctuations indicate the period from lower low-water to higher highwater (rising tide) and from higher highwater to lower low-water (falling tide), respectively. CTD profiling measurements of (**b**) salinity, (**c**) temperature, and (**d**) density are contoured, respectively. The white lines in (c) indicate 21.9 °C and 22 °C isotherms. The black dots above the CTD data denote times of profiling. The periods with onshore current are marked with the black rectangles. Circled numbers "1" and "2" indicate the examples of the NIWs described in the text and in following figures.

To understand the tidal motions in the upper part of the water column, the stick diagram of vectorially averaged tidal velocity above the 5-m depth is plotted against the fluctuations of water elevation measured by the ADCP on the mooring (Fig. 3a). At the mooring location, because of diurnal inequality, tidal flows were landward during one (or two) consecutive rising tides (from lower low-water to higher highwater, marked “R”) and seaward during one (or two) consecutive falling tide (from higher highwater to lower low-water, marked "F"). Each rising and falling cycle included two floods and ebbs. The tidal range was around 2-m, which is defined as a micro- to meso- tidal environment^49,50^.

### Water-column structures

The hydrographic profiling revealed temporal variation in the water-column structure at the ZHJ2 (Fig. 3b–d). The vertical salinity values ranged between 28.6 psu near the surface and 34.6 psu near the bottom. The vertical temperature structure changed from 30 °C at the surface to 21.9 °C near the bottom. The sigma-t values ranged from 18 to 24.15 kg/m^3^. Because of the salinity and temperature structures, the density field displayed strong vertical density gradients especially at around 10 m and showed the lowest water density at the surface (lowest salinity also) at end of experiment.

The above vertical structures differentiate two types of regimes. The upper water column was occupied by warm, less saline water sourced from the Pearl River effluent. Due to the on-offshore tidal oscillations, the plume regime was modulated with alternating water masses of low salinity, high temperature (during rising tide; marked with the rectangles) or high salinity, low temperature (during falling tide). Furthermore, the surface water was rapidly displaced downward occasionally for short durations (e.g. selected examples marked "1" and "2" in Fig. 3b). In the lower water column, a secular trend of decreasing temperature existed and is perceived by the depth of the 22 °C isotherm from 10 to 20 m above bed. Punctuated by the tidal current, the colder water signals showed pulses (Fig. 3c).

### Mixing parameter (S_p_)

**Figure 4:**
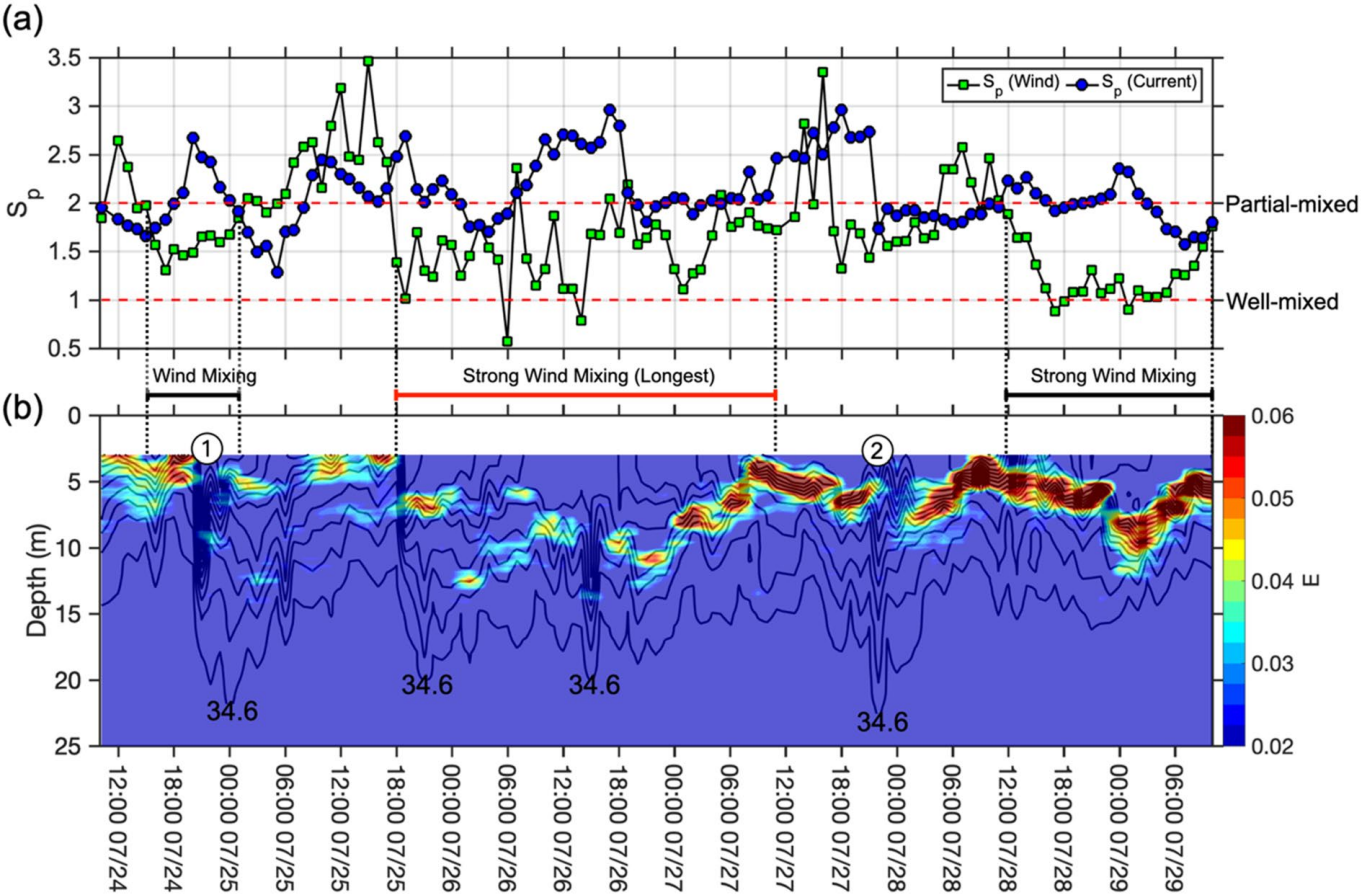
The temporal variability of stratification parameters at ZHJ2. (**a**) The S_p_ induced by wind (green squares) and current (blue circles). The red dashed lines indicate S_p_ equals to 1 and 2, which stand for the threshold of well-mixed and partial mixing in the water column. The black and red bars below the S_p_ graph mark the periods of strong wind mixing. (**b**) The color contours of the static stability index, E, at ZHJ2. Salinity contours (black lines) are superimposed. Circled numbers indicate the examples of the NIWs described in the text and in following figures.

The extent of boundary-related mixing to the water column was evaluated by the mixing parameter S_p_ (Fig. 4a). The wind-induced S_p_ and the current-induced S_p_ were calculated to quantify the contributions of wind and current in the surface layer (below 3 m). When S_p_ > 2, the water column is well stratified. When S_p_ < 1, the water column is well mixed.

The values of S_p_ induced by currents were mostly around or greater than 2 during the experiment at ZHJ2. This implies that bottom friction drag on the current-generated mixing was insufficient to mix the entire water column^21^. However, around 79% of the wind-induced S_p_ through air-sea boundary was smaller than 2. This suggests the wind contributed sufficiently to the mixing force in the surface layer especially during the period between 18:00 July 25th and 11:00 July 27th (marked by a red bar). It is noted that this period was the longest when the S_p_ values were continuously lower than 2.

### Stratification parameter (E)

The Static Stability Index (E) quantified the degree of water-column stratification. The positive value of E signifies water-column stability. Conversely, negative E denotes unstable water column. There was a layer of high positive E values in the upper water column (Fig. 4b) and formed a dynamic barrier preventing vertical mixing as described by previous works^21,51^. However, the depth of high positive E values fluctuated vertically, and E values had occasional gaps. The gaps most frequently occurred in periods of low E values and favorable wind mixing when S_p_ were lower than 2 marked by the red bar (Fig. 4). Additionally, comparisons of E and salinity profiles indicated the stability gaps co-existed with surface plume water drawdowns (representative examples marked "1" and "2"; Figs. 3b, 4b).

### Propagation of internal solitary waves from northern South China Sea to ZHJ2

**Figure 5:**
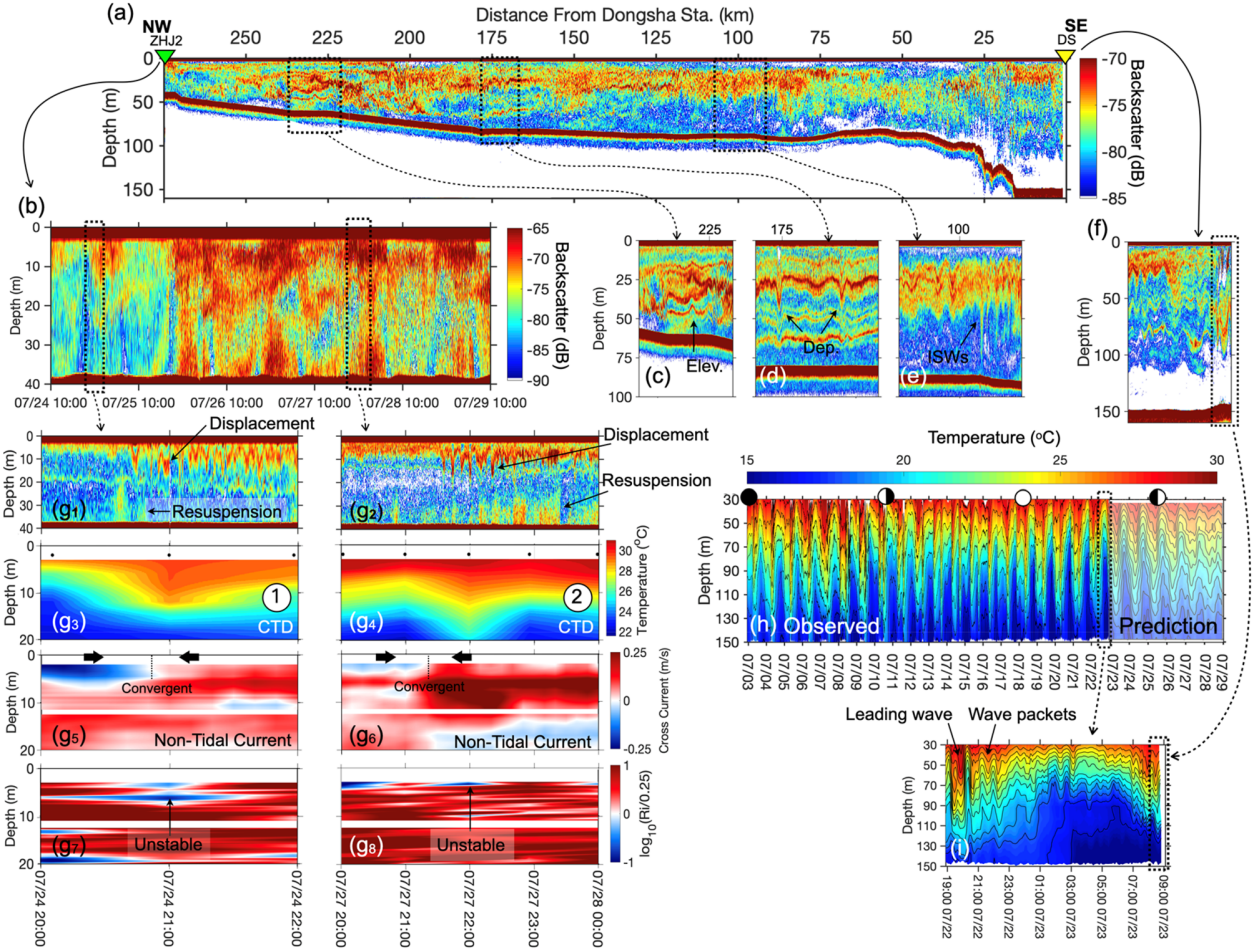
The shoaling processes of the NIWs observed in the northern South China Sea. (**a**) The echogram recorded by EK60 along the transect between DS and ZHJ2 shown in Fig. 1. (**b**) The temporal variability of the acoustic backscatter profiles measured by R/V OR-1 at ZHJ2 during the experiment. (**c**) to **(f)** enlarged segments of the echogram transect in (**a**). (**g**_**1-2**_) are the enlarged segments in the temporal acoustic backscatter at two different time slots indicated by black dashed rectangles in (**b**). (**g**_**3-4**_) are the synchronized temperature profiles recorded by CTD for the same periods as (**g**_**1-2**_) which are marked by the circled numbers 1, 2 in previous figures. The black dots above the temperature contours denote the times of profiling. (**g**_**5-6**_) are contoured cross-shore non-tidal currents, in which red color is landward, and blue color is seaward. (**g**_**7-8**_) are contoured Richardson number. The red color indicates the stable condition, and the blue indicates the turbulence regime. (**h**) The temporal variation of the temperature structure in the water column measured at DS. The white circles with black in portion indicate the period from new moon to full moon. The translucent part in (h) indicates the predicted temperature variation by harmonic analysis. (**i**) Is the enlarged segment of (h) at the time when the last two NIWs passed before retrieving the mooring at the DS. The time marked with black dashed rectangle corresponds to the black dashed rectangle marked on the spatial echogram (f).

Using the echo sounder, EK60, typical NIWs are recognized along the transect from DS to the ZHJ2 (Fig. 5a–f). The echograms provide a strong evidence that the energy of the NIWs were propagating at depth approximately 25 m from the NSCS into the Guangdong coastal area. During the shoaling process, the NIWs transitioned from depression waves to elevation waves between the depth range of 75 to 100 m (also indicates the critical depth), which were 175 to 225 km away from DS (Fig. 5c,d). The results show the amplitudes of depression wave and elevation wave were around 25 m and 10 m, respectively. This deformation of shoaling NIW was also found at nearshore of Dongsha Atoll by previous studies^10,52–54^.

The thermometers mounted on the mooring at DS displayed that the NIW frequently appeared at the end of July in 2016 (Fig. 5h–i) were followed by wave packets. According to harmonic analysis results, the NIWs presented in diurnal periods that were mainly composed of K_1_ and O_1_ tidal components (Table 1). The ER was less than 50% in depths shallower than 55 m possibly because of the nonlinear effect by the passing NIWs. Since the NIW is considered to be driven by the barotropic tide^4,55^, the harmonic tidal analysis was also used to predict the arrivals of the NIW at Sta. DS until the end of experiment, and the results indicate the NIWs consistently occur diurnally (Fig. 5h).

At ZHJ2, the echogram shows that the energy of NIWs presented as the wave packets with 6–8 h duration in the upper water column (Fig. 5b,g1,g2, Supplementary Fig. 4). As wave packets arrived, the vertical water movement was perturbed, and the maximum downward displacement was 20 m. Simultaneously, the bottom sediment resuspension occurred. The NIW patterns were also recorded by CTD and current profiles. The surface warm water was drawn down as the convergent current occurred, causing the ‘sandwich structure’ in the F and ER above 7 m (Figs. 3b–d, 5g3–6, Table 1). This convergent current pattern was similar to the observation offshore New Jersey^12^. The low Ri values (Ri/0.25 < 1) indicated the water became unstable when the surface warm water was drawn down (Fig. 5g7–8). Afterwards, the increasing Ri value indicated the stability returned when the warm water was restored to the surface layer. However, it should be noted that since the CTD and current profiling were taken at hourly and 10-min intervals, respectively, potential aliasing could exist with capturing higher frequency NIWs. Therefore, the presence of NIWs revealed in the hydrographic and current data here should be used in a qualitative manner.

## Discussion

**Figure 6:**
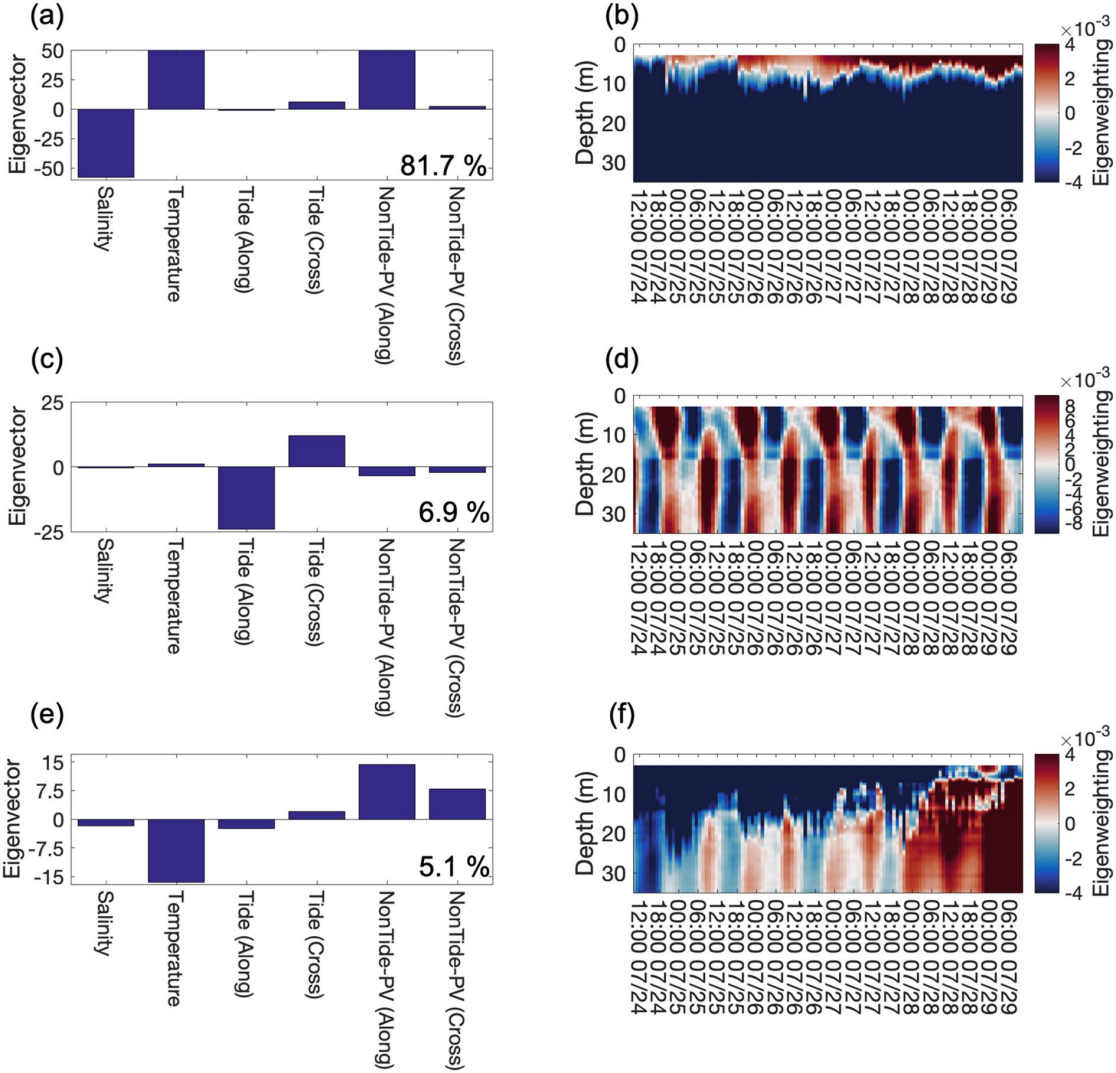
The eigenvectors and contoured eigenweightings of each eigenmode. (**a**) and (**b**) show the eigenvectors and contoured eigenweightings of 1st eigenmode. The positive contoured eigenweightings indicate the co-variability shown in the eigenvector. The negative eigenweightings indicate the inversed correlations shown in the eigenvector. (**c**) and (**d**) show the eigenvectors and contoured eigenweightings of 2nd eigenmode. (**e**) and (**f**) show the eigenvectors and contoured eigenweightings of 3rd eigenmode.

To further decipher the process-response relations among tide, non-tidal forcing (e.g. wind), and water-column responses (salinity and temperature), a multivariate analysis tool, Empirical Orthogonal Function (EOF) analysis, was used. The tidal and non-tidal forcing were represented by the along and across-shore components in order to clarify the water-column responses to the hydrodynamic processes. The EOF analysis decomposed above spatial/vertical and temporal co-varying variables into independent (orthogonal) modes that bear separate process-response patterns in the data set (Fig. 6).

The 1st eigenmode explains 81.7% of the correlations (standardized co-variance), and its eigenvectors show salinity co-varied inversely to temperature and non-tidal current in the alongshore component (Fig. 6a). This indicates the fresher and warmer river plume water (negative salinity and positive temperature) with the northeastward non-tidal current (positive non-tidal alongshore component) dominated the co-variability in this mode. Since the surface current direction was connected with the wind direction (Supplementary Fig. 5), this mode describes the transport of the river plume by the northeastward wind-driven flow, which is the most important factor to influence the co-variability of the entire data set.

The contour plot of the eigenweightings of the 1st mode shows the temporal pattern of the river plume signature in the vertical structure (Fig. 6b). The line of zero crossings of the eigenweightings indicates the partition between buoyant river-plume regime at the surface and the coastal water regime underneath^21,23^. The depth of this line varied between 5 and 10 m, which delineates the lower boundary of the river plume influence. The spikes on the partition indicate water mass perturbations that is not resolved by this mode.

The influence of tides was resolved by the 2nd eigenmode (Fig. 6c,d), which explains 6.9% of the correlations. The sign of the tidal components mainly suggests the dominance of NW–SE-ward tidal flows (inversely between alongshore and across-shore tidal components). The temporal variability of vertical eigenweighting structures shows strong periodicities (Fig. 6d). During the early part of the flood tide (from lower low-water to lower highwater; periods marked with "F1" in Supplementary Fig. 6), the strong landward tidal flows dominated the entire water column at diurnal intervals. During the successive ebb tide (marked with "E1"), the alongshore diurnal tidal flows dominated the water column, whose strength decreased with depth.

Additionally, the eigenweightings showed a phase offset in the tidal flow between the upper and lower water column at the depth around 15 m. The landward tidal component (positive eigenweighting) presents the diurnal period near the surface, but semi-diurnal period in the lower layer. Since the river plume regime accounts for 81.7% of the correlations, the influence of the tide was ‘blurred’. Subsequently, the co-variability due to the river plume regime of the 1st eigenmode was removed from the original dataset^56^. EOF was again used on the residual dataset. The result enhanced the tidal influence. The diurnal and the semi-diurnal tides account for 24.1% and 43.6% of the residual correlations, respectively (Supplementary Fig. 7). The water column shallower than 15 m was dominated by diurnal-dominated mixed tide, which was composed of K_1_ tide mainly with NE-SW orientation (Fig. 2e and Table 1). On the other hand, deeper than 15 m, the tidal current was semidiurnal-dominated mixed tide, which was comprised of M_2_ tide with NW–SE current direction. The frequency analysis was also used to provide additional information of the data, and results are shown in Supplementary Fig. 8.

The 3rd eigenmode of the original dataset explains 5.1% of the correlations (Fig. 6e). The eigenvectors show the water temperature were grouped inversely to alongshore and cross-shore nontidal flows. Since alongshore non-tidal current is regarded as wind-driven (Fig. 2, Supplementary Fig. 5), this mode demonstrates the cold-water intrusion related to the wind, which leads to upwelling near the coast^37,42,43^. The contour plot of eigenweightings indicates the thickening of the cold water in the lower column that gradually extended upward into shallower depths (the red patch in Fig. 6f). Ultimately, the intrusion reached 20 m above bed. The eigenweightings also depict that the water temperature fluctuated at semi-diurnal frequencies between 06:00 July 25 and 12:00 July 27, which corresponded to the across-shore tidal currents (Figs. 3c,6f).

The EOF analysis clearly established a hierarchy in the process-response pattern at a fixed point on the pathway of the Pearl River plume. Firstly, as the fresher and warmer Pearl River effluent exited the PRE, the typical southwesterly summer monsoon and northeastward wind-induced current were the primary forcing to cause the river plume to disperse northeastward and seaward (Supplementary Figs. 1 and 2). Then, the Pearl River effluent is propagating along the Guangdong coast and enters the Taiwan Strait^36,42,57^. On this setting, the presence of the buoyant river plume caused strong stratification in the upper part of the water column along the river plume dispersal pathway (between 5 and 10 m depth defined by EOF; Figs. 3b–d, 4b, 6a,b). The stratification created a density barrier against water exchange between river plume water and ambient seawater beneath (Fig. 4). Above the stratified layer, the mixing was induced by wind because the low S_p_ indicates the prevailing wind-mixed environment. The wind induced mixing could disrupt the in-situ stratification occasionally when the water density gradient was low^21,27^ (marked as the red bar in Fig. 4b).

The disruption in the water stratification was also caused by the vertical water displacement that entrained the buoyant plume water downward abruptly that appeared as the distinctive pulses (marked "1" and "2" in Figs. 3b, 4b, 5g1–8). Eventually, turbulence was generated (Ri < 0.25) and caused temporal gaps in the stratification. Our observations imply that the events of water displacements corresponded to the passing NIWs, which were propagating from the northern SCS basin westward to offshore PRE (Fig. 5). This interpretation is also supported by the satellite images that indicate internal wave fronts propagate shoreward on the shelf in the northern SCS^58,59^ (Supplementary Figs. 9, 10).

The NIW-induced water displacements occurred at diurnal frequencies offshore PRE (Figs. 3b–d, 4b, 5g), because NIWs from the SCS remained in the tidal signals along the propagating pathway^53^. After the NIW-induced mixing energy dissipated, the water-column stability returned. Since the stratification prevents vertical exchange between the river plume water and ambient seawater, the NIW-induced perturbations could contribute to mixing processes among different water masses offshore PRE. To the best of our knowledge, our study is the first occasion to observe the influence of far-field NIWs on the river plume dynamics and local mixing processes. Although other studies pointed out that mechanisms (e.g. the plume-related hydraulic jump and tidal pumping etc.) might trigger the similar phenomenon^31,60–62^, our measurements (e.g. strong across-shore current variation, consistently NE wind; Figs. 2a and 5g5–6) demonstrates that the perturbating energy was from the offshore.

Tidal motions, which were the second most important forcing, resulted in the diurnal and semi-diurnal fluctuations in the signature of the buoyant river plume (Fig. 3). Flooding tide pushed the buoyant plume landward, and ebbing tide pushed the plume water seaward. Thus, noticeable temporal variability in the vertical structures of salinity and temperature occurred, and water stratification fluctuated diurnally. Similar mechanisms have been discussed in river plumes of the Changjiang River and Columbia Rivers^41,63,64^.

Although tidal processes only account for small percentage of the total co-variability in the dataset, it is by no means that tidal forcing was less complex in the hydrodynamic mechanisms. After removing the 1^st^ eigenmode component from the data, the tidal processes explained 67.7% of the residual co-variability. The results indicated the vertical partition of the tidal influence was around 15-m depth. Above this depth, the diurnal tide, K_1_, dominated the tidal forcing; below this depth, the semi-diurnal tide, M_2_, controlled the tidal forcing (Figs. 2e, 6c,d, and Supplementary Figs. 7, 8).

The tertiary mechanism was the cold-water intrusion in the lower water column related to the wind-driven coastal upwelling (Figs. 3c, 6e,f). Throughout the study period, the vectorially averaged southwesterly winds were upwelling favorable^37^ (Fig. 2a). Therefore, the offshore water flowed northwestward near the bottom (Fig. 2b), leading to the intrusion of cold water near bottom (Fig. 3c). The thickness of the bottom water mass with vertically homogeneous temperature structure, defined by the depth of the 22 °C isotherm, which gradually moved upward in the water column (Figs. 3c, 6f). However, when the wind direction changed to non-upwelling favorable conditions due to the influence of the Tropical Storm Mirinae, the bottom current started to stagnate, and the intrusion of the offshore water paused (Figs. 2a,c, 3c). Within this period, diurnal and semi-diurnal tidal currents became the dominant factor to modulate the bottom water-mass movements (Figs. 3c, 6f). The intrusion resumed with the increasing magnitude of northeastward current as the southwesterly wind started blowing again. This implies that the contributions of the wind and tidal forcing to changing the bottom water mass property cannot be neglected and should be considered when evaluating biogeochemical mechanisms for coastal hypoxia at the nearby Mirs Bay^29,65^. Although previous studies have reported that the prevailing southwesterly wind induced a net landward bottom flow by using modelling^37,43,47^, our research provides the temporal variation of bottom current filed and its response to the wind with *in-situ* measurement.

In addition to the process-response hierarchy, the EOF analysis also revealed vertical partition between adjacent regimes at discrete depths. The boundary between the wind-driven river plume and ambient shelf regimes existed between 5 and 10 m depth. Within the tidal regime, a partition appeared at about 15-m depth in both the semi-diurnal and diurnal frequencies. At this point, the mechanism for this partition is subject to future studies. The last partition is between the cold-water intrusion regime near the seafloor and the overlying ambient shelf regime. During our study, the boundary of this regime moved toward the surface, suggesting increased flux of cold-water mass.

**Figure 7:**
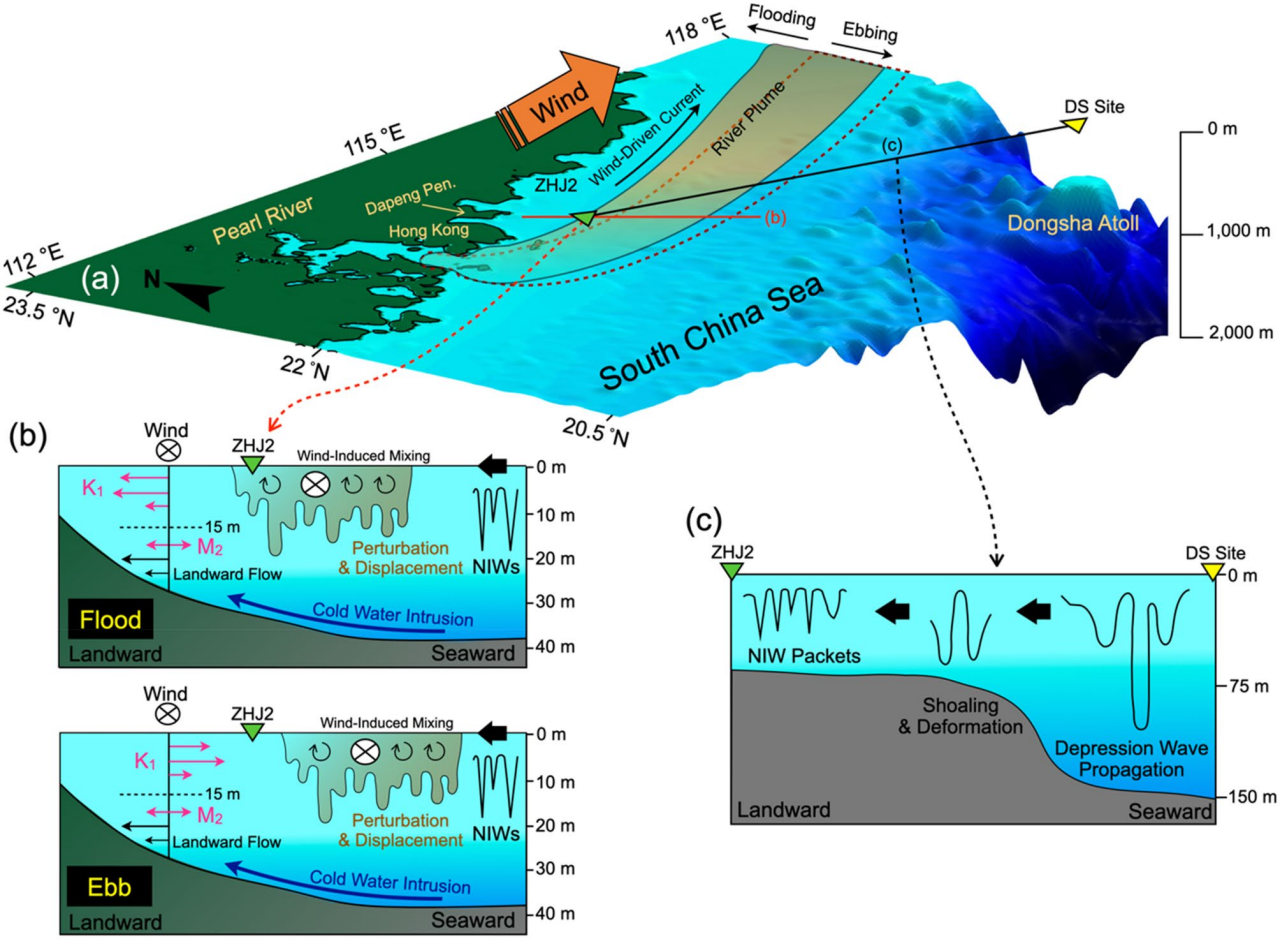
The schematic diagram describing the coupling mechanisms. (**a**) shows the coupling mechanisms among the wind, river plume dispersal, and tidal motions, and (**b**) the cross-section shows the mechanisms including wind and tidal mixings, NIW-induced perturbation, and cold water intrusion. The upper and lower panels show the period during flood and ebb, respectively. (**c**) The landward propagation of NIWs from the South China Sea between DS and ZHJ2 showing transformation from elevation waves to depression waves. The bold arrows indicate the direction of the NIW propagation along the orientation of the 2-D sections.

Major findings of this study are illustrated in a schematic diagram in Fig. 7. The northeastward currents driven by upwelling favorable monsoon winds dispersed the Pearl River plume in the NE direction and triggered the intrusion of cold-water mass near the bottom (Fig. 7a,b). The buoyant plume water created strong stratification at the surface, which set the ‘stage’ for other physical processes. Since tidal currents caused the plume water mass to swing landward in the flood and seaward in the ebb, it influenced the thickness of the plume water mass and stratified layer (Fig. 7b). The wind field also could increase the thickness of the stratified layer and disrupt the water stability occasionally when the density gradient was low in the water column^21,27^ (red bar marked in Figs. 4b, 7b). Furthermore, in some instances, the stratification in the river plume regime was disrupted by the shoreward propagating NIWs from the northern SCS (Fig. 7c). The NIWs created perturbations to draw surface water downward (Fig. 7b). Our findings provide a new insight into how the NIWs influence the mixing processes in the far field of the river plume dispersal. This could improve our understanding of the transport and dispersal processes of the terrestrial material sourced from the PRE to the oceanic sink in NSCS shelf and Taiwan Strait.

## Methods

### Shipboard observations

Two cruises were carried out by using R/V Ocean Researcher III on June 3rd—5th at ZHJ1 and R/V Ocean Researcher I on July 24th—29th at ZHJ2 for shipboard monitoring and mooring deployment and retrieval (Fig. 1a). ZHJ1 was located in the Dangan Channel in which the water depth was around 30 m. ZHJ2 was on the open shelf, 98 km NE away from the PRE, where the water depth was about 45 m.

At ZHJ1, the 75 kHz shipboard ADCP was used to obtain the current field with 1-min sampling interval and 0.12 m/s precision. The first bin of shipboard ADCP was at 16.5 m with 8-m bin size. At ZHJ2, hourly hydrographic profiling was conducted by using Seabird SBE 19 plus CTD with 0.2-m bin size. Along a transect from DS to ZHJ2 (insert in Fig. 1a), the acoustic backscatter strength profile was recorded by SIMRAD EK60 echo sounder (38 kHz) with 1-s sampling interval and 0.4-m bin size to capture the propagating NIWs in the northern SCS. Meteorological data including atmospheric pressure, wind speed and direction were recorded at 10-min intervals by the weather station onboard the R/Vs.

### Mooring deployments

A 44.7-m taut-line mooring was deployed nearby ZHJ2 (Fig. 1a,b) on July 24th—29th. The mooring was configured with Teledyne RDI 1200 kHz ADCP (upward-looking at 11 m below the surface) and Teledyne RDI 300 kHz ADCP (downward-looking at 12.7 m). The bin-sizes for two ADCPs were 0.5-m (1200 kHz) and 1-m (300 kHz) with blank distance 0.5-m and 1-m, respectively. The velocity resolution both for 1200 kHz and 300 kHz ADCP was 0.1 cm/s. The precision of the pressure sensor mounted on the ADCP was 0.01 decibar. The sampling intervals for all instruments were synchronized at 10-min intervals.

Another 150-m long taut-line mooring was deployed at Station DS (117.248°E, 21.8432°N; Fig. 1a,c), north of Dongsha Atoll, near the Sta. S7 conducted by Ramp et al.^53^. The mooring was mounted with 4 DST milli-TDs (marked T/P; thermometer with the pressure sensor) and 8 8-bit mini-logs (marked T; thermometer) at 27.8, 46.7, 57.6, 68.8, 91.9, 103.9, 116.2, 128.7, 141.6, and 150.7 m depths, respectively. The sampling rate of each sensor was 6-min to capture the signature of the passing NIWs between June 3rd and July 23rd. The accuracy of pressure measured by DST milli-TD is ± 1.5 m, and the accuracy of temperature recorded by the 8-bit mini-log is ± 0.3 °C.

### Hydrodynamic analysis and mixing indicators

The instantaneous current field is divided into two components, non-tidal current and tidal current, in this study. The tidal components in the current data were resolved by using T_Tide (http://www.eos.ubc.ca/~rich/t_tide/t_tide_v1.3beta.zip), a MATLAB program to perform the harmonic analysis.

1 $$V_{p} \left( t \right) = \mathop \sum \limits_{k = 1}^{m} \alpha_{k} (\cos \omega_{k} t - \theta_{k} )$$

where ${\mathrm{V}}_{\mathrm{p}}$ is the predicted tidal current including barotropic and baroclinic tide, m is the number of tidal components used in the analysis; ${\mathrm{\alpha }}_{\mathrm{k}}$ is the amplitude of the kth tidal component; ${\upomega }_{\mathrm{k}}$ and ${\uptheta }_{\mathrm{k}}$ indicate the tidal angular frequency and corresponding phase lag, respectively; ${\upomega }_{\mathrm{k}}= 2\uppi /{\mathrm{T}}_{\mathrm{k}},$ where ${\mathrm{T}}_{\mathrm{k}}$ is the period of the kth tidal component; t is the sampling time. In this study, only tidal components of which the signal to noise ratio (SNR) higher than 2 were used. The detailed information about calculation of SNR is described by Pawlowicz et al.^66^.

The tidal-to-total energy ratio (ER) in the current was calculated to quantify the energy contribution of the synthetic tidal flow to the instantaneous flow^67^ and defined as:

2 $${\text{ER}} = \frac{{\sum\nolimits_{{t = 1}}^{n} {\left( {{\text{V}}_{{\text{p}}} (t) - \overline{{{\text{V}}_{{\text{p}}} }} } \right)} ^{2} }}{{\sum\nolimits_{{t = 1}}^{n} {\left( {{\text{V}}(t) - \overline{{\text{V}}} } \right)^{2} } }} \times 100\%$$

where n is the length of the data series. ${\bar{{{\mathrm{V}}}}_{\mathrm{p}}}$ is the mean tidal current velocity.${\bar{{\mathrm{V}}}}$ is the instantaneous current velocity. When the ER value is lower, it indicates the contribution to fluctuating the current field by the tidal forcing is weaker.

The form number, F, is used to identify the type of the tidal environment. It is defined as the amplitude ratio between major diurnal tidal and semi-diurnal tidal components.

3 $$\text{F = }\frac{\mathrm{K}1+\mathrm{O}1}{\mathrm{M}2+\mathrm{S}2}$$

F = 0 ~ 0.25: Semi-diurnal tide.

F = 0.25 ~ 1.5: Mixed tide with semi-diurnal dominance.

F = 1.5 ~ 3: Mixed tide with diurnal tide dominance.

F > 3: Diurnal tide.

The static stability index (E) was calculated to quantify the water column stability^21,23,51,68^. The water column is stable when E is positive and unstable when E is negative.

4 $$\text{E = -}\frac{1}{{\uprho }_{0}}\left(\frac{{\partial}{\uprho}_{\text{w}}}{{\partial z}}\right)$$

where ${\uprho }_{0}$ is average water density in the measurement, ${\partial\uprho }_{\mathrm{w}}$ and $\partial$ z is the vertical gradient of the water density and the water depth. The z-axis is positive upward. The buoyancy frequency is also calculated and shown in Supplementary Fig. 11 as the reference.

The stratification parameter, S_p_, was used to reveal the relative contribution of tidal mixing and wind mixing in the water column^69^. S_p_ is expressed as:

5 $${{\text{S}}_{\text{p}}} = {\log _{10}}\frac{1}{\varepsilon }$$

when S_p_ > 2, is well stratified, and when S_p_ < 1, the water column is well-mixed. $\upvarepsilon$ stands for the energy dissipation rate per unit mass, and $\upvarepsilon$ induced by wind and current can be expressed as:

6 $$\varepsilon _{{{\text{wind}}}} = \frac{{10^{4} \times \rho _{{\text{a}}} {\text{C}}_{{10}} {\text{K}}\left| {{\text{U}}_{{10}} } \right|^{3} }}{{\rho _{0} z}}$$

7 $$\varepsilon _{{{\text{current}}}} {\text{ = }}\frac{{10^{4} \times {\text{C}}_{{\text{D}}} \left| {\overline{{{\text{U}}_{{\text{p}}} }} } \right|^{3} }}{{\text{h}}}$$

where ${\uprho }_{\mathrm{a}}$ is the air density (kg/m^3^), C_10_ (~ 0.0016) is the drag coefficient for the wind at 10 m height, K is the wind factor (~ 0.004), U_10_ is the wind speed at 10 m height, ${\uprho }_{0}$ is the average water density in the measurement, and z is the water depth at specific layer, and 3 m was used in this study.${\mathrm{C}}_{\mathrm{D}}$ is the bottom drag coefficient (~ 0.0025), and ${\bar{{{\mathrm{U}}}}_{\mathrm{p}}}$ is the depth-average tidal velocity (m/s), h is the water depth from bottom to the specific layer (41.7 m used in this study).

The mixing mechanism induced by the passing NIWs was assessed by the Richardson number, Ri. Ri value greater than 0.25 indicates the stable environment; otherwise unstable as the turbulence overcomes stratification. The Ri shown in Fig. 5g7, 8 was divided by 0.25 to normalize the value.

8 $${\text{R}}_{\text{i}}\text{=}\frac{{\text{N}}^{2}}{{\text{S}}^{2}}$$

where $\mathrm{N}$ is the buoyancy frequency, $\mathrm{N}$ is expressed as $\mathrm{N}=\sqrt{\mathrm{gE}}$. g stands for the gravity acceleration. ${\mathrm{S}}^{2}$ is the current-induced vertical shear velocity in the horizontal dimension and is expressed as

9 $${\text{S}}^{2}\text{=}\left(\frac{{\partial u}}{{\partial z}}\right)^{2}\text{+}{\left(\frac{{\partial v}}{{\partial z}}\right)}^{2}$$

where u and v are current velocities in E-W and N-S component, respectively.

### EOF (empirical orthogonal/eigen function) analysis

EOF, a widely used statistical tool for multivariate analysis^70^, was used to decompose the synoptic data of co-varying variables in the water column affected by multiple factors into different orthogonal (independent) modes with particular sets of eigenvectors, eigenvalues, and eigenweightings. The higher the eigenvalue of an eigenmode is, the more co-variability among parameters this mode explains. In this study, salinity and temperature measured at each cast were regarded as the river plume variables to determine water column variability caused by the interplay between plume hydrodynamics and physical processes^21,23,24^.

## Supplementary Information

Supplementary Information.

## Data Availability

The measurement data during the cruises is available as .mat files on request to the corresponding author. The MODIS satellite images were available from the data portal (https://worldview.earthdata.nasa.gov/).

## References

[1] Masunaga E, Arthur RS, Fringer OB, Yamazaki H (2017). Sediment resuspension and the generation of intermediate nepheloid layers by shoaling internal bores. J. Mar. Syst..

[2] Rayson MD, Ivey GN, Jones NL, Fringer OB (2018). Resolving high-frequency internal waves generated at an isolated coral atoll using an unstructured grid ocean model. Ocean Model..

[3] Sandstrom H, Elliott JA (1984). Internal tide and solitons on the scotian shelf: a nutrient pump at work. J. Geophys. Res..

[4] Alford MH (2015). The formation and fate of internal waves in the South China Sea. Nature.

[5] Wang YH, Dai CF, Chen YY (2007). Physical and ecological processes of internal waves on an isolated reef ecosystem in the South China Sea. Geophys. Res. Lett..

[6] Munk W, Wunsch C (1998). Abyssal recipes II: Energetics of tidal and wind mixing. Deep Sea Res..

[7] Tuerena RE (2019). Internal tides drive nutrient fluxes into the deep chlorophyll maximum over mid-ocean ridges. Glob. Biogeochem. Cycles.

[8] Lucas AJ (2011). The green ribbon: Multiscale physical control of phytoplankton productivity and community structure over a narrow continental shelf. Limnol. Oceanogr..

[9] Lucas AJ, Franks PJS, Dupont CL (2011). Horizontal internal-tide fluxes support elevated phytoplankton productivity over the inner continental shelf. Limnol. Oceanogr. Fluids Environ..

[10] Fu KH, Wang YH, St. Laurent L, Simmons H, Wang DP (2012). Shoaling of large-amplitude nonlinear internal waves at Dongsha Atoll in the northern South China Sea. Cont. Shelf Res..

[11] Shishkina OD, Sveen JK, Grue J (2013). Transformation of internal solitary waves at the ‘deep’ and ‘shallow’ shelf: Satellite observations and laboratory experiment. Nonlin. Process. Geophys..

[12] Shroyer EL, Moum JN, Nash JD (2009). Observations of polarity reversal in shoaling nonlinear internal waves. J. Phys. Oceanogr..

[13] Cai S, Xie J (2010). A propagation model for the internal solitary waves in the northern South China Sea. J. Geophys. Res. Ocean..

[14] Cai S, Xie J, He J (2012). An overview of internal solitary waves in the South China Sea. Surv. Geophys..

[15] Zhao Z (2014). Internal tide radiation from the Luzon Strait. J. Geophys. Res. Ocean..

[16] Jia Y (2019). Deep-sea sediment resuspension by internal solitary waves in the Northern South China Sea. Sci. Rep..

[17] Pineda J (1994). Internal tidal bores in the nearshore: Warm-water fronts, seaward gravity currents and the onshore transport of neustonic larvae. J. Mar. Res..

[18] van Haren H (2012). Internal wave turbulence near a texel beach. PLoS ONE.

[19] Hetland RD (2005). Relating river plume structure to vertical mixing. J. Phys. Oceanogr..

[20] Jurisa JT, Nash JD, Moum JN, Kilcher LF (2016). Controls on turbulent mixing in a strongly stratified and sheared tidal river plume. J. Phys. Oceanogr..

[21] Du X, Liu JT (2017). Particle dynamics of the surface, intermediate, and benthic nepheloid layers under contrasting conditions of summer monsoon and typhoon winds on the boundary between the Taiwan Strait and East China Sea. Prog. Oceanogr..

[22] Horner-Devine AR, Hetland RD, MacDonald DG (2014). Mixing and transport in coastal river plumes. Annu. Rev. Fluid Mech..

[23] Lee J, Liu JT, Hung C-C, Lin S, Du X (2016). River plume induced variability of suspended particle characteristics. Mar. Geol..

[24] Liu JT (2019). Three-dimensional coupling between size-fractionated chlorophyll-a, POC and physical processes in the Taiwan Strait in summer. Prog. Oceanogr..

[25] Wright, L. D. River deltas BT: Coastal sedimentary environments. (ed. Davis, R. A.) 1–76 (Springer, New York, 1985). 10.1007/978-1-4612-5078-4_1.

[26] Glenn SM (2016). Stratified coastal ocean interactions with tropical cyclones. Nat. Commun..

[27] Scully ME, Friedrichs C, Brubaker J (2005). Control of estuarine stratification and mixing by wind-induced straining of the estuarine density field. Estuaries.

[28] Susanto RD, Pan J, Devlin AT (2019). Tidal mixing signatures in the Hong Kong coastal waters from satellite-derived sea surface temperature. Remote Sens..

[29] Zhang H, Cheng W, Chen Y, Yu L, Gong W (2018). Controls on the interannual variability of hypoxia in a subtropical embayment and its adjacent waters in the Guangdong coastal upwelling system, northern South China Sea. Ocean Dyn..

[30] Nash JD, Moum JN (2005). River plumes as a source of large-amplitude internal waves in the coastal ocean. Nature.

[31] Osadchiev AA (2018). Small mountainous rivers generate high-frequency internal waves in coastal ocean. Sci. Rep..

[32] Arnoux-Chiavassa S, Rey V, Fraunié P (1999). Modelling of suspended sediment fluxes off the rhône river mouth. J. Coast. Res..

[33] Wright LD, Nittrouer CA (1995). Dispersal of river sediments in coastal seas: Six contrasting cases. Estuaries.

[34] Chao SY, Boicourt WC (1986). Onset of estuarine plumes. J. Phys. Oceanogr..

[35] Liu JT, Chao S, Hsu RT (2002). Numerical modeling study of sediment dispersal by a river plume. Cont. Shelf Res..

[36] Bai Y (2015). Intrusion of the Pearl River plume into the main channel of the Taiwan Strait in summer. J. Sea Res..

[37] Chen Z, Pan J, Jiang Y, Lin H (2017). Far-reaching transport of Pearl River plume water by upwelling jet in the northeastern South China Sea. J. Mar. Syst..

[38] Gan J, Li L, Wang D, Guo X (2009). Interaction of a river plume with coastal upwelling in the northeastern South China Sea. Cont. Shelf Res..

[39] Huang TH (2019). East China Sea increasingly gains limiting nutrient P from South China Sea. Sci. Rep..

[40] Wang J, Hong H, Jiang Y, Chai F, Yan XH (2013). Summer nitrogenous nutrient transport and its fate in the Taiwan Strait: A coupled physical-biological modeling approach. J. Geophys. Res. Ocean..

[41] Zu T, Gan J (2015). A numerical study of coupled estuary-shelf circulation around the Pearl River Estuary during summer: Responses to variable winds, tides and river discharge. Deep. Res..

[42] Ou S, Zhang H, Wang DX (2009). Dynamics of the buoyant plume off the Pearl River Estuary in summer. Environ. Fluid Mech..

[43] Chen Z, Jiang Y, Liu JT, Gong W (2017). Development of upwelling on pathway and freshwater transport of Pearl River plume in northeastern South China Sea. J. Geophys. Res. Ocean..

[44] Gan J, Cheung A, Guo X, Li L (2009). Intensified upwelling over a widened shelf in the northeastern South China Sea. J. Geophys. Res. Ocean..

[45] Wong LA (2003). A model study of the circulation in the Pearl River Estuary (PRE) and its adjacent coastal waters: 1. Simulations and comparison with observations. J. Geophys. Res..

[46] Zhang Q, Xu CY, Zhang Z (2009). Observed changes of drought/wetness episodes in the Pearl River basin, China, using the standardized precipitation index and aridity index. Theor. Appl. Climatol..

[47] Wang D (2014). Relative contributions of local wind and topography to the coastal upwelling intensity in the northern South China Sea. J. Geophys. Res. Ocean..

[48] Saylor JH (1994). Studies of bottom ekman layer processes and mid-lake upwelling in the laurentian great lakes. Water Qual. Res. J..

[49] Short A (1991). Macro-meso tidal beach morphodynamics: An overview. J. Coast. Res..

[50] Wang J (2018). The coupling of bay hydrodynamics to sediment transport and its implication in micro-tidal wetland sustainability. Mar. Geol..

[51] Liu JT (2018). A comprehensive sediment dynamics study of a major mud belt system on the inner shelf along an energetic coast. Sci. Rep..

[52] Fu K-H, Wang Y-H, Lee C-P, Lee I-H (2016). The deformation of shoaling internal waves observed at the dongsha atoll in the Northern South China Sea. Coast. Eng. J..

[53] Ramp SR, Yang YJ, Bahr FL (2010). Characterizing the nonlinear internal wave climate in the northeastern South China Sea. Nonlinear Process. Geophys..

[54] Reeder DB, Ma BB, Yang YJ (2011). Very large subaqueous sand dunes on the upper continental slope in the South China Sea generated by episodic, shoaling deep-water internal solitary waves. Mar. Geol..

[55] Apel JR (1997). An overview of the 1995 SWARM shallow-water internal wave acoustic scattering experiment. IEEE J. Ocean. Eng..

[56] Wu CR, Chang CWJ (2005). Interannual variability of the South China Sea in a data assimilation model. Geophys. Res. Lett..

[57] Hu J (2011). Variable temperature, salinity and water mass structures in the southwestern Taiwan Strait in summer. Cont. Shelf Res..

[58] Huang, W., Johannessen, J., Alpers, W., Yang, J. & Gan, X. Spatial and temporal variations of internal waves in the northern South China Sea. *2nd Int. Work. Adv. SAR Oceanogr. from ENVISAT ERC Mission. January* 21–25 (2008).

[59] Wang J, Huang W, Yang J, Zhang H, Zheng G (2013). Study of the propagation direction of the internal waves in the South China Sea using satellite images. Acta Oceanol. Sin..

[60] Honegger DA, Haller MC, Geyer WR, Farquharson G (2017). Oblique internal hydraulic jumps at a stratified estuary mouth. J. Phys. Oceanogr..

[61] Walter RK, Phelan PJ (2016). Internal bore seasonality and tidal pumping of subthermocline waters at the head of the Monterey submarine canyon. Cont. Shelf Res..

[62] Walter RK, Stastna M, Woodson CB, Monismith SG (2016). Observations of nonlinear internal waves at a persistent coastal upwelling front. Cont. Shelf Res..

[63] Horner-Devine AR, Jay DA, Orton PM, Spahn EY (2009). A conceptual model of the strongly tidal Columbia River plume. J. Mar. Syst..

[64] Wu H, Zhu J, Shen J, Wang H (2011). Tidal modulation on the Changjiang River plume in summer. J. Geophys. Res. Ocean..

[65] Li XL, Shi HM, Xia HY, Zhou YP, Qiu YW (2014). Seasonal hypoxia and its potential forming mechanisms in the Mirs Bay, the northern South China Sea. Cont. Shelf Res..

[66] Pawlowicz R, Beardsley B, Lentz S (2002). Classical tidal harmonic analysis including error estimates in MATLAB using TDE. Comput. Geosci..

[67] Hsu M-H, Kuo AY, Kuo J-T, Liu W-C (1999). Procedure to calibrate and verify numerical models of estuarine hydrodynamics. J. Hydraul. Eng..

[68] Knauss JA (1978). Introduction to Physical Oceanography.

[69] Pingree RD, Holligan PM, Mardell GT (1978). The effects of vertical stability on phytoplankton distributions in the summer on the northwest European Shelf. Deep. Res..

[70] Hannachi A, Jolliffe IT, Stephenson DB (2007). Empirical orthogonal functions and related techniques in atmospheric science: A review. Int. J. Climatol..

