# Supplementary material for: Encountering shoaling internal waves on the dispersal pathway of the pearl river plume in summer
Source: Sci Rep. 2021 Jan 13;11:999. doi: 10.1038/s41598-020-80215-2 (PMC7807090; doi:10.1038/s41598-020-80215-2)
Supplement: Supplementary file 1 — Supplementary Information. [file 41598_2020_80215_MOESM1_ESM.pdf]

Supplementary Information for:

Encountering Shoaling Internal Waves on the Dispersal Pathway of the Pearl River plume in Summer

Jay Lee[1], James T. Liu[1*], I-Huan Lee[1], Ke-Hsien Fu[1,2], Rick J. Yang[1], Wenping Gong[3], Jianping Gan[4]

[1]Department of Oceanography, National Sun Yat-sen University, Kaohsiung, Taiwan R.O.C.

[2]Marine Science and Information Research Center, National Academy of Marine Research, Kaohsiung, Taiwan R.O.C.

[3]School of Marine Sciences, Sun Yat-sen University, Guangzhou, Guangdong, China

[4]Department of Ocean Science & Department of Mathematics, School of Science, The Hong Kong University of Science and Technology, Kowloon, Hong Kong

*Corresponding author

Correspondence to james@mail.nsysu.edu.tw

Tel: +886-7-525-5144, Fax: +886-7-525-5130

Contents

Supplementary Figure 1: The meteorological background and current field measured at ZHJ1 in June, 2016.

Description of Supplementary Figure 1

Supplementary Figure 2: The higher frequency stick diagram of current profile measured by 300 kHz and 1200 kHz ADCP deployed at ZHJ2.

Supplementary Figure 3: The PV graph of instantaneous flow at ZHJ2 with 1-m interval color codes.

Supplementary Figure 4: The echograms recorded by EK60 at ZHJ2.

Supplementary Figure 5: Wind field direction vs. surface current direction (≤1.6 m vectorially averaged) measured at ZHJ2.

Supplementary Figure 6: The comparison between the mean tidal current and contoured eigenweightings of 2nd eigenmode.

Supplementary Figure 7: The diagram to show the EOF results after removing the component of the 1st eigenmode (river plume component) from the original dataset.

Supplementary Figure 8: The frequency analysis for alongshore and cross-shore current

Supplementary Figure 9: The portion of the Terra MODIS satellite images corresponding to the study area.

Supplementary Figure 10: The distribution of internal wave shown in Fig. 4a by Wang et al. (2013).

Supplementary Figure 11: The static stability (E) and buoyancy frequency (N^2) profilers in the experiment.

Supplementary references

Supplementary Figure 1:

The meteorological background and current field measured at ZHJ1 in June, 2016.

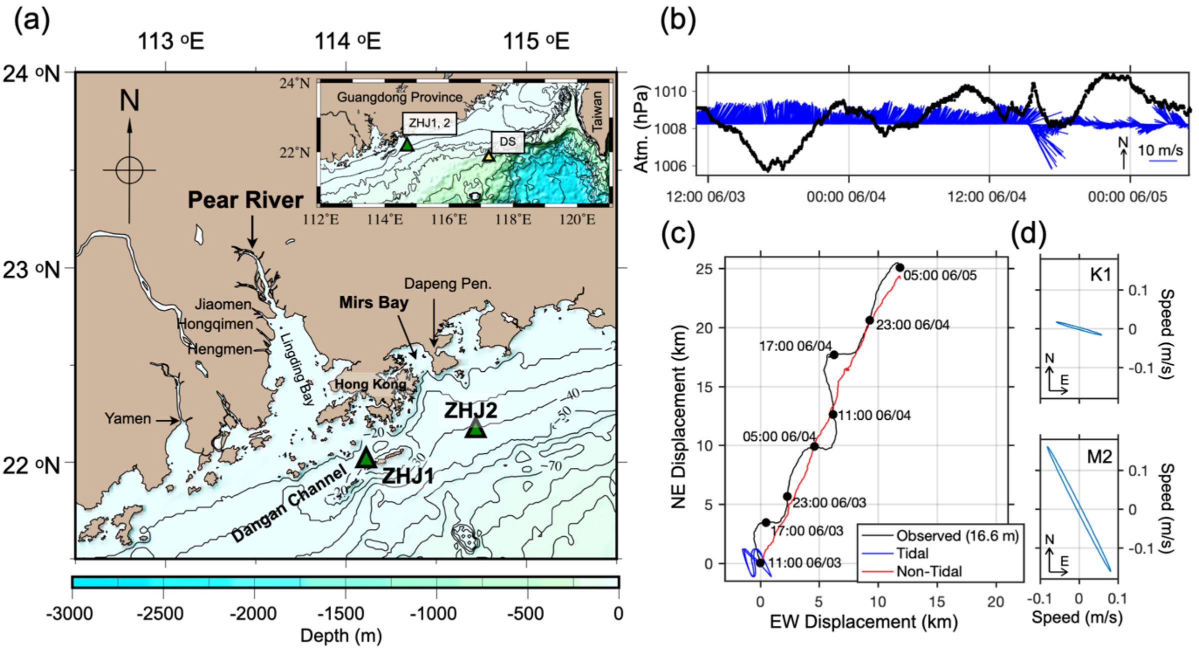

(a) The map plotted using the Generic Mapping Tools (GMT, V5.4.2, https://www.soest.hawaii.edu/gmt/) shows the locations the two monitoring sites (green triangles) in June (ZHJ1) and July (ZHJ2) of 2016. **(b)** The stick diagram of the wind field and atmospheric pressure measured aboard the research vessels at ZHJ1. The stick diagrams are plotted according to the oceanographic convention. The north is the upward direction. Both the wind and pressure fields were plotted at 20-min interval. **(c)** the progressive vector diagram of the instantaneous current (thick black curve), the tidal (thin blue curve), and non-tidal (thin red curve) components measured at 16.6 m depth at ZHJ1. **(d)** are tidal current ellipses for K_1 and M_2 tides.

Description of Supplementary Figure 1

The wind field at ZHJ1 was plotted as the stick diagram in oceanographic convention (Supplementary. Fig. 1b). The steady prevailing southerly/southwesterly winds dominated the wind field in the first 24 hours of the record (average speed 5.87 m/s), and the winds turned easterly after a 2-hour transition period to northwesterly winds. The atmospheric pressure gradually increased during the observation. However, the increase was more noticeable when the winds turned easterly.

Superimposed by the M_2 and K_1 tidal components on the non-tidal flow (Supplementary. Fig. 1c, d), the instantaneous current was largely flowing a zigzag pattern in northwest-southeast directions (on-offshore) and along the Dangan Channel (Supplementary. Fig. 1a, b). Near the end of the observation, the tidal signal in the instantaneous current became weak, and the current direction remained consistently northeastward. The average speed of the instantaneous current in the measurement was 0.18 m/s.

Supplementary Figure 2:

The higher frequency stick diagram of current profile measured by 300 kHz and 1200 kHz ADCP deployed at ZHJ2.

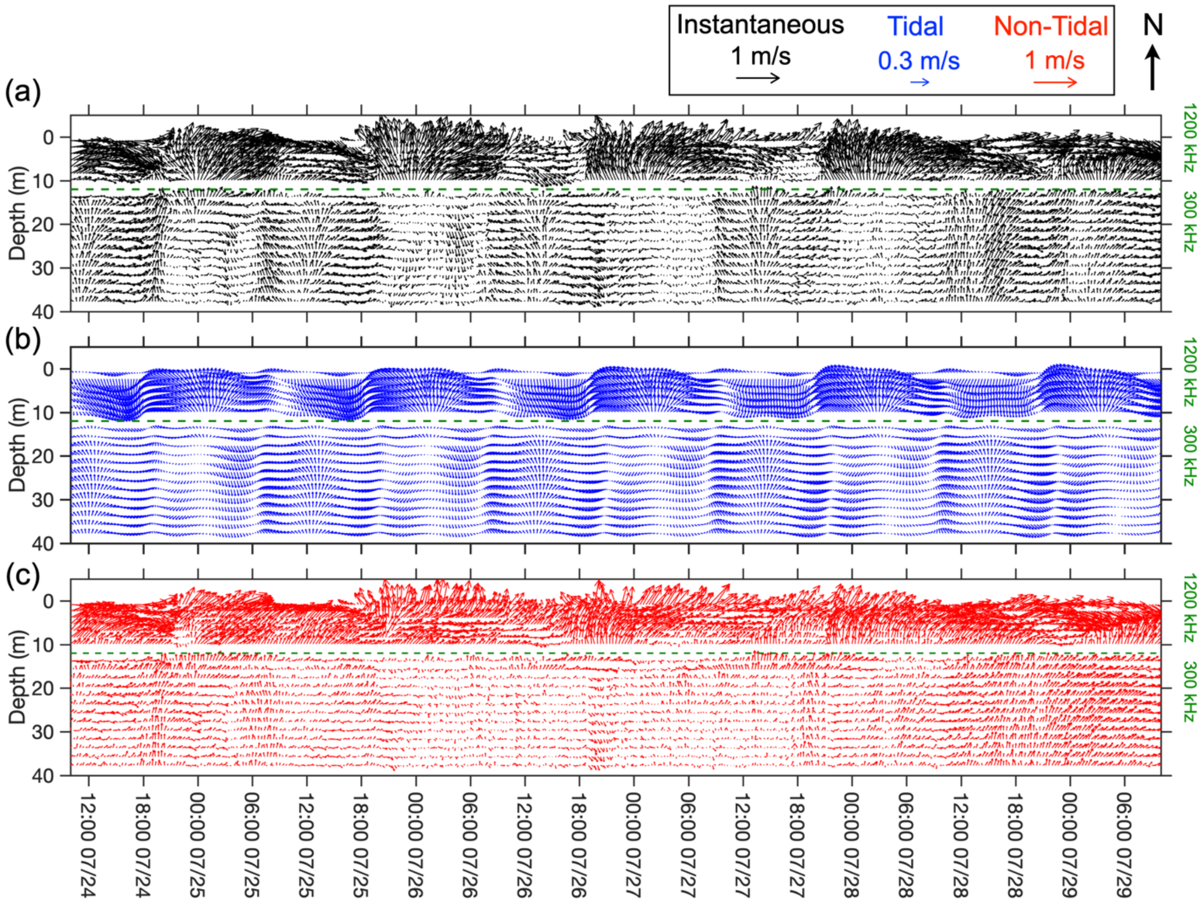

(a) to **(c)** indicate the instantaneous flow, tidal, and non-tidal components, respectively. The green dash lines indicate the depth at which the ADCPs were deployed. The north is the upward direction. The stick diagrams were plotted at 10 mins interval.

Supplementary Figure 3:

The PV graph of instantaneous flow at ZHJ2 with 2-m interval color codes.

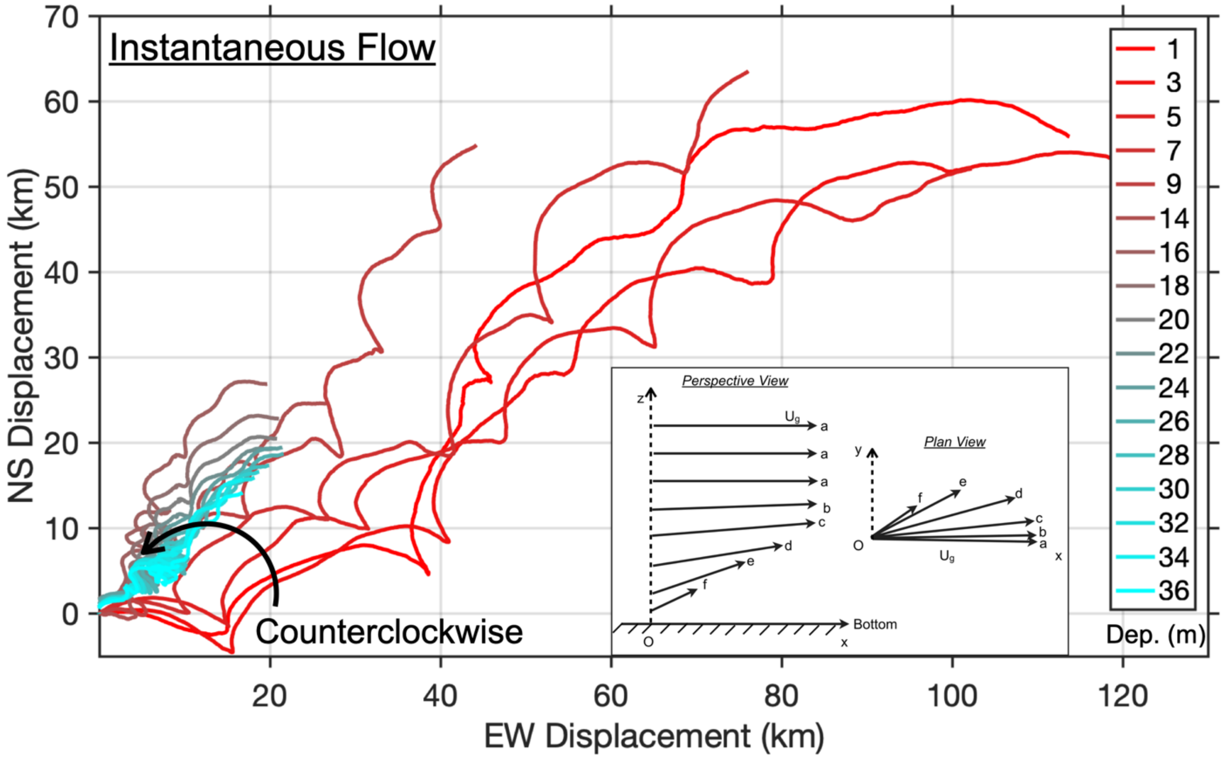

This figure describes the current structure influenced by the bottom friction with the conceptual definition (Saylor, 1994).

Supplementary Figure 4:

The echograms recorded by EK60 at ZHJ2.

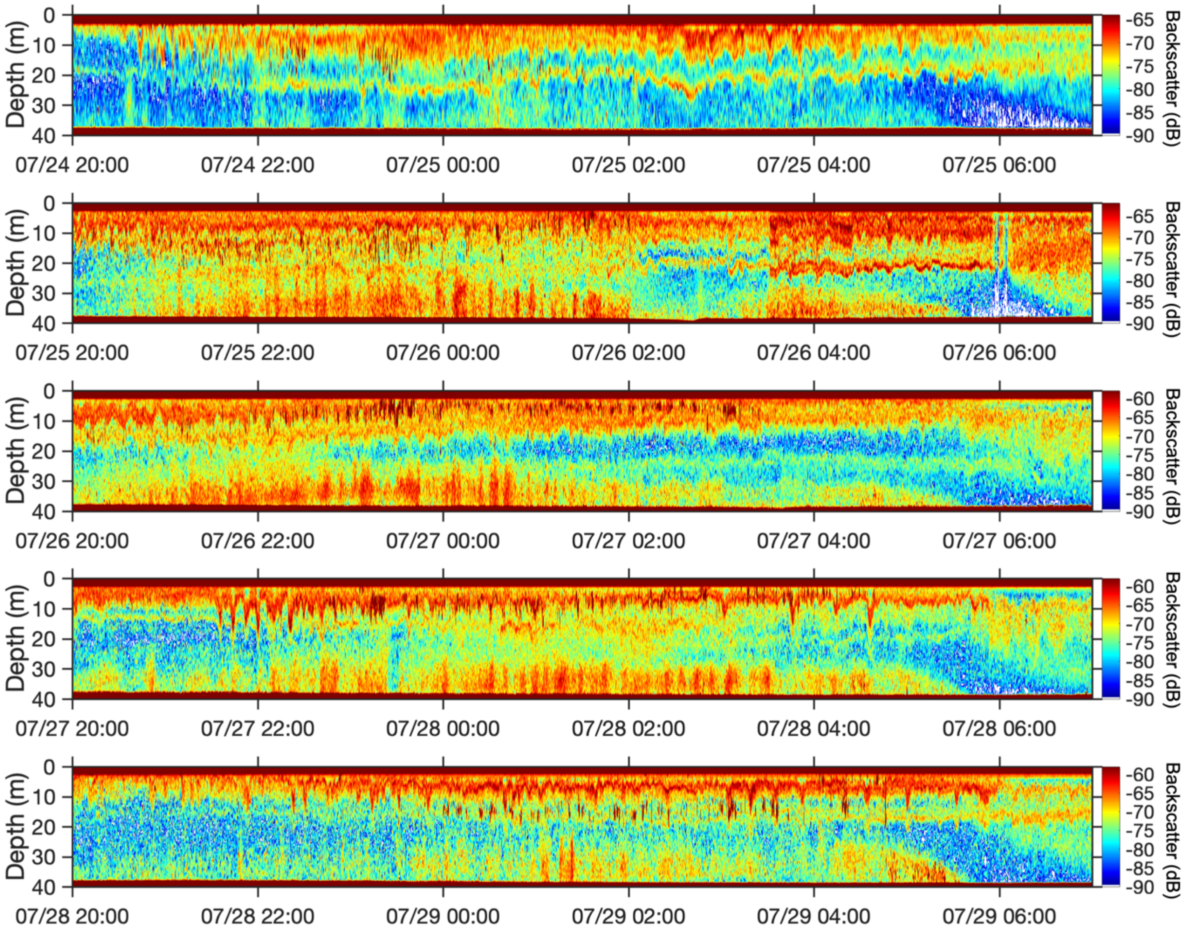

From the top to the bottom showing the 12-hour acoustic backscatter measurements during the experiment.

Supplementary Figure 5:

Wind field direction vs. surface current direction (≤1.6 m vectorially averaged) measured at ZHJ2.

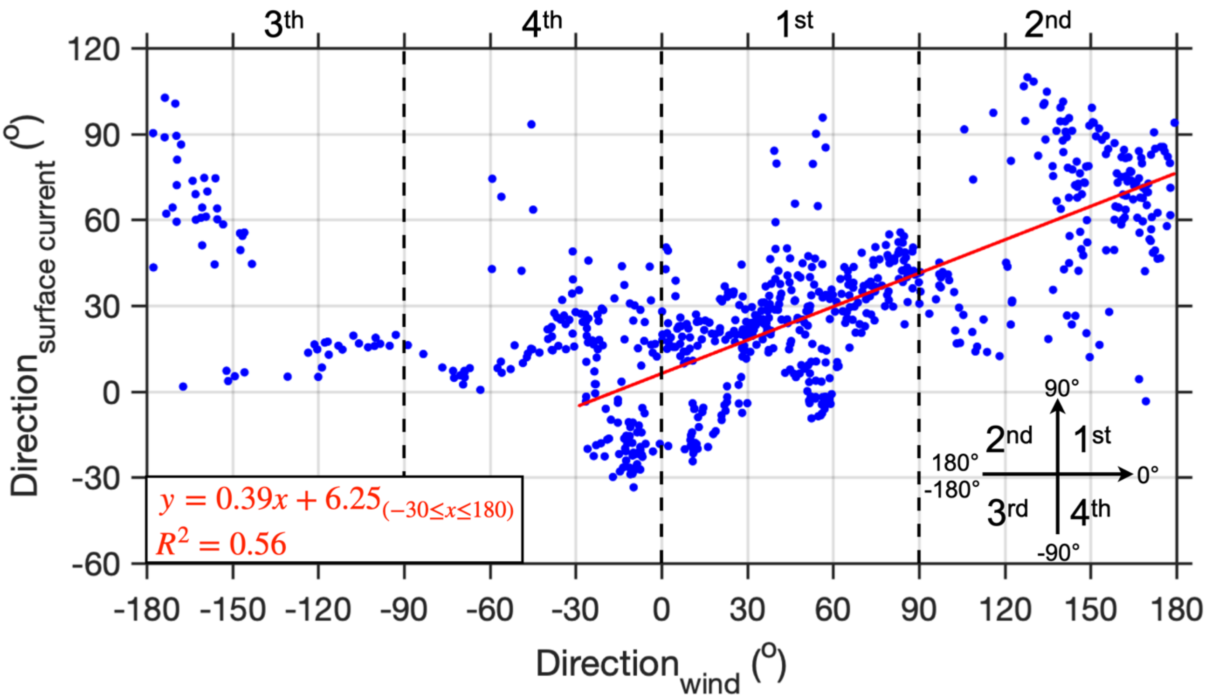

The linear regression line between these two variables is expressed as a red line with the linear equation and R squared value. Black dash lines labeled with numbers indicate the quadrants showed in the insert at lower right. The 1st quadrant is defined from 0° to 90°; 2nd quadrant is defined from 90° to 180° (-180°); 3rd quadrant is defined from -180° to -90°; 4th quadrant is defined from -90° to 0°.

Supplementary Figure 6:

The comparison between the mean tidal current and contoured eigenweightings of the 2nd eigenmode.

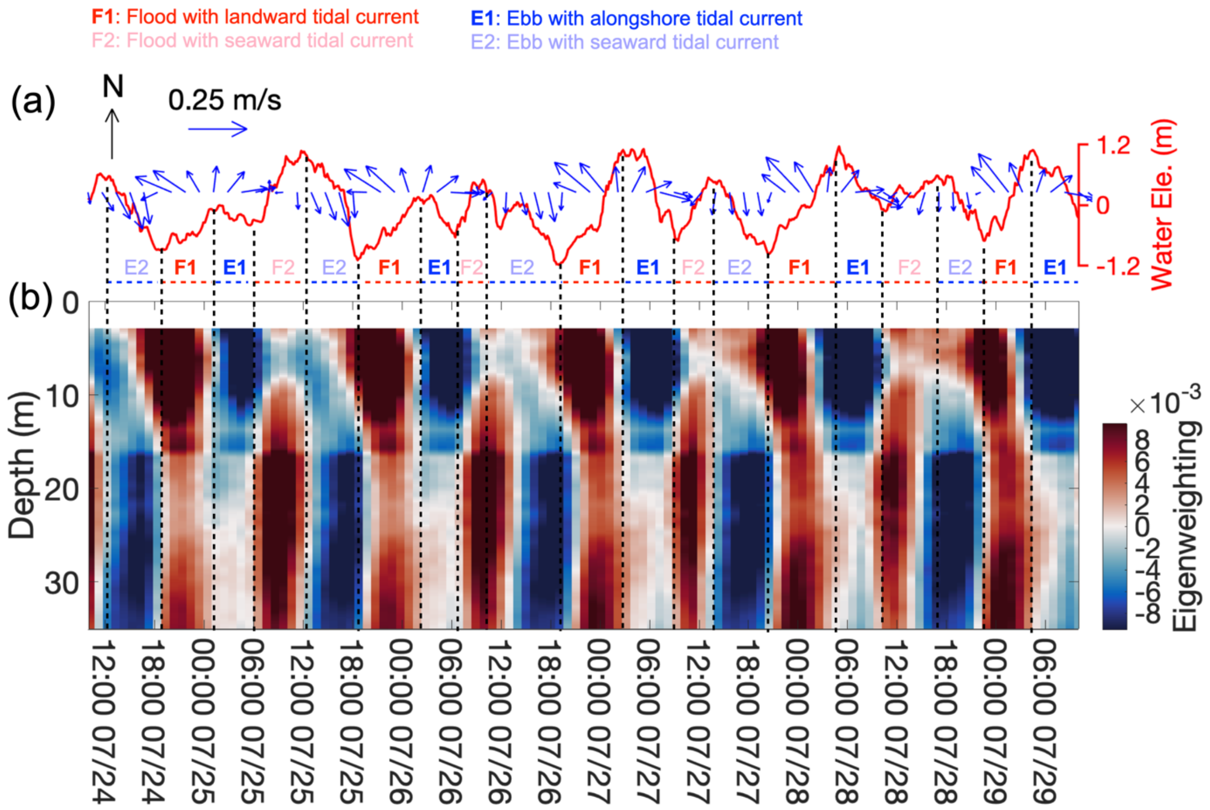

(a) is same as the Fig. 3a. This stick diagram shows the mean tidal current averaged over the top 5 m superimposed with the sea-surface fluctuations (red line) recorded by the ADCP. The **(b)** is same as Fig. 6d, which is contoured eigenweightings of the 2nd eigenmode. F1 and F2 indicate the landward and seaward tidal currents during the flood, respectively. E1 and E2 indicate the alongshore and seaward tidal currents during the ebb, respectively.

Supplementary Figure 7:

The diagram to show the EOF results after removing the component of the 1st eigenmode (river plume component) from the original dataset.

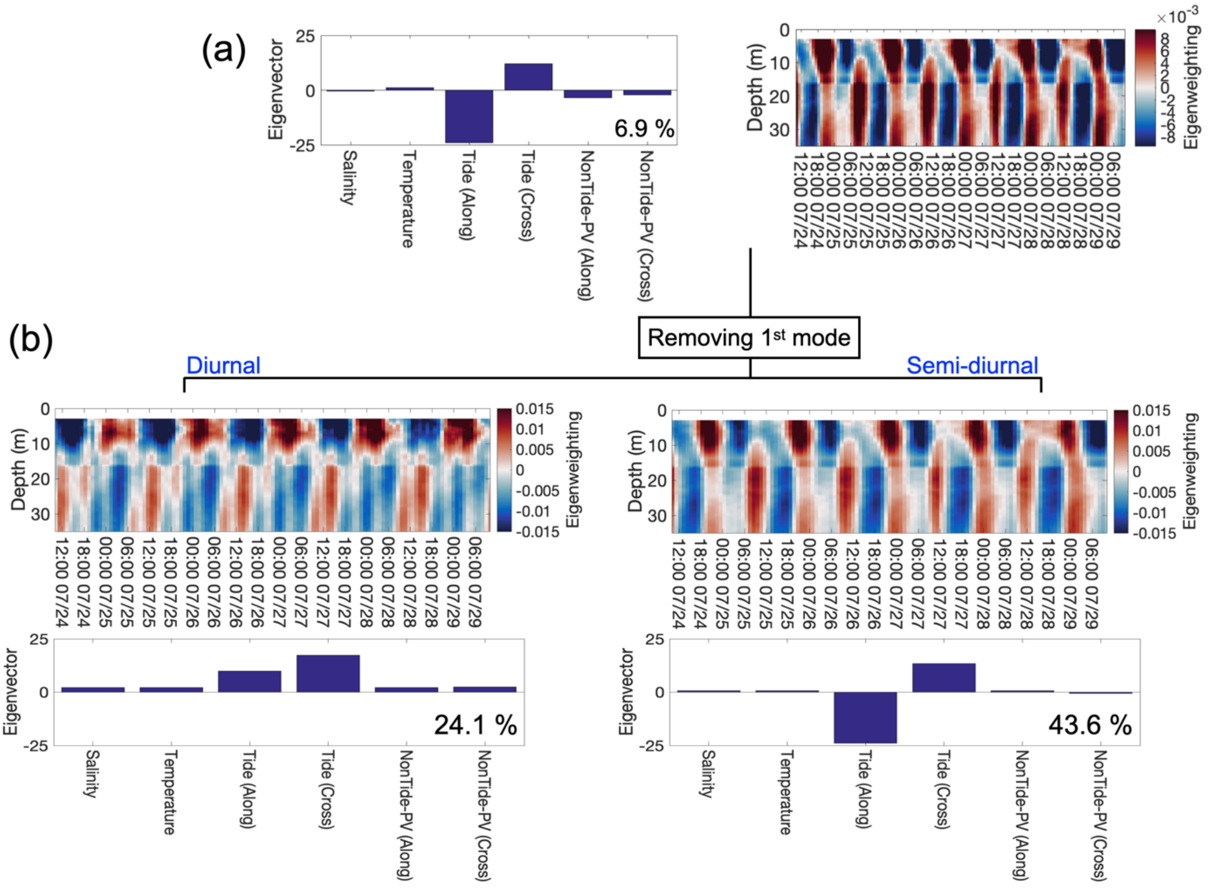

(a) is same as the Fig. 6c, d. **(b)** shows the of the eigenweighting and eigenvector of the first two eigen modes without the influence of the river plume component.

Supplementary Figure 8:

The frequency analysis for alongshore and cross-shore current

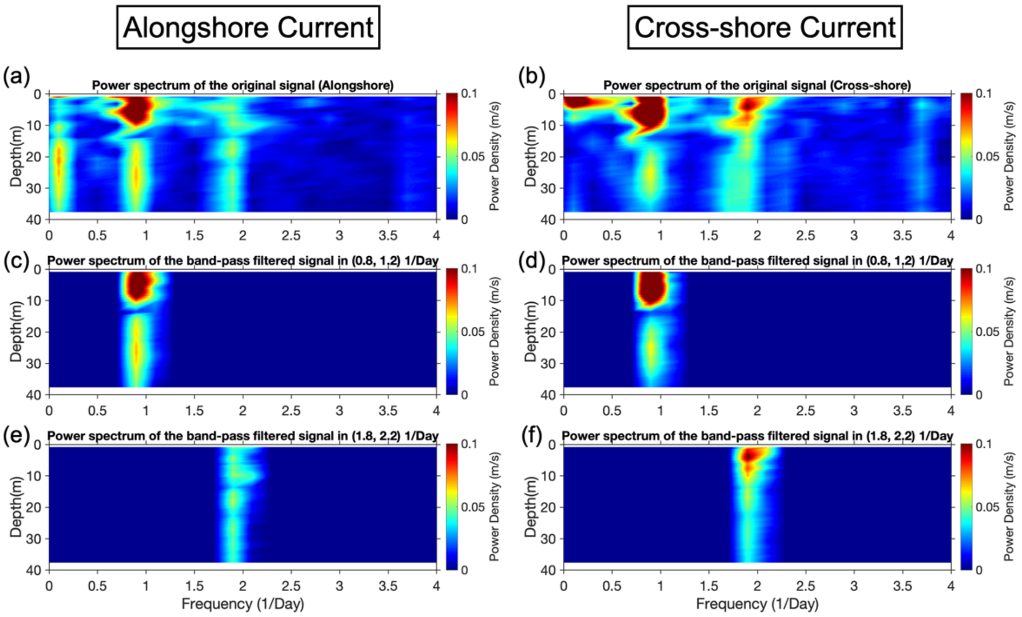

(a-b) show the power spectrum of the original data. **(c-d)** show the power spectrum with the band-pass filter between 0.8 to 1.2 per day. **(e-f)** show the power spectrum with the band-pass filter between 1.8 to 2.2 per day.

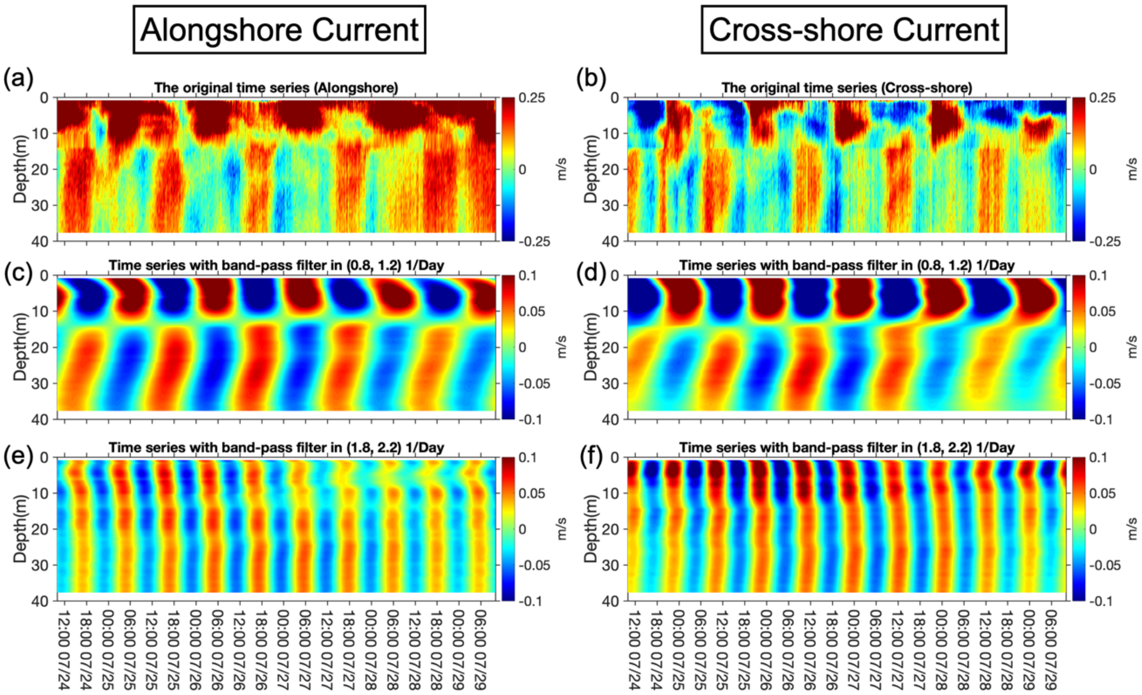

(a-b) The original alongshore and cross-shore current profiling. **(c-d)** show alongshore and cross-shore current profilers after filtering by the band-pass filter between 0.8 to 1.2 per day. **(e-f)** show the alongshore and cross-shore current profiling after filtering by the band-pass filter between 1.8 to 2.2 per day.

Supplementary Figure 9:

The portion of the Terra MODIS satellite images corresponding to the study area.

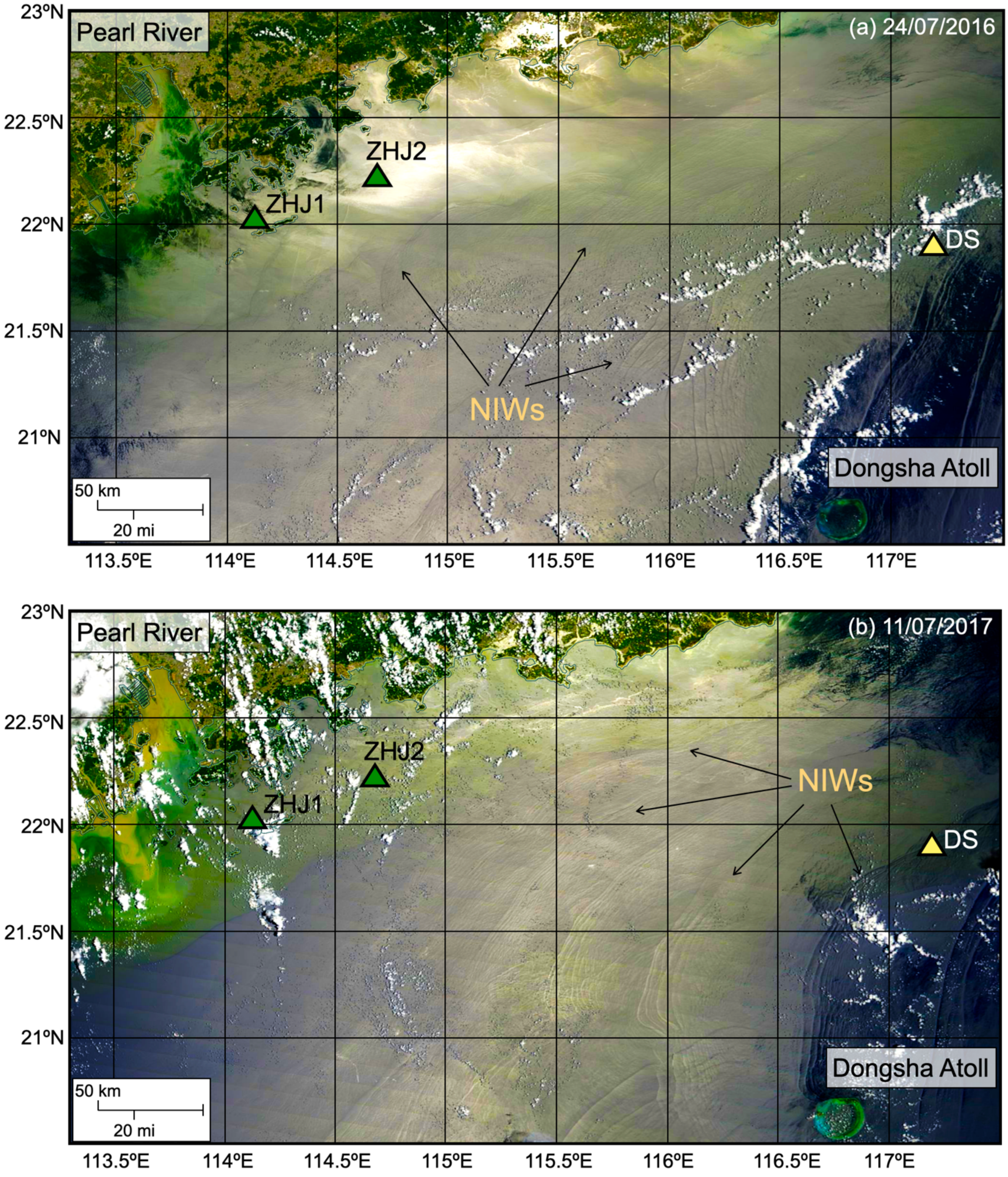

(a) Image captured in July 24th, 2016 (b) Images captured in July 11th, 2017. The satellite images were captured by NASA Worldview (https://worldview.earthdata.nasa.gov/). The positions of NIWs are labeled. The green and yellow triangles indicate the study sites shown in Fig. 1.

Supplementary Figure 10:

The distribution of internal wave shown in Fig. 4a by Wang et al. (2013).

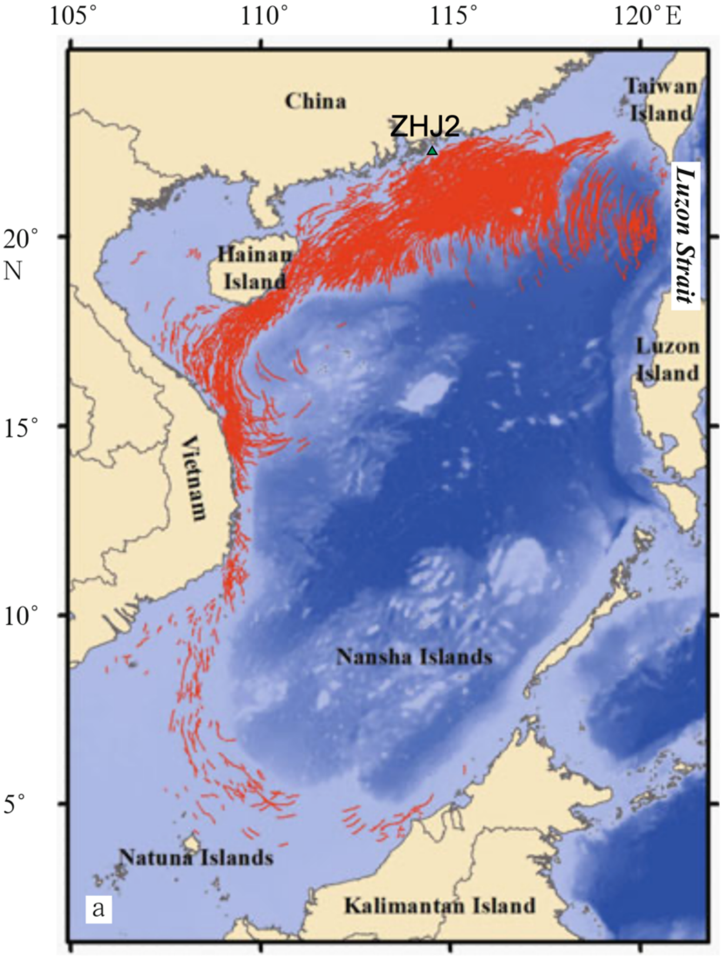

Red lines indicate the internal waves distributing in the South China Sea. The green triangle is ZHJ2 site.

Supplementary Figure 11:

The static stability (E) and buoyancy frequency (N^2) profilers in the experiment

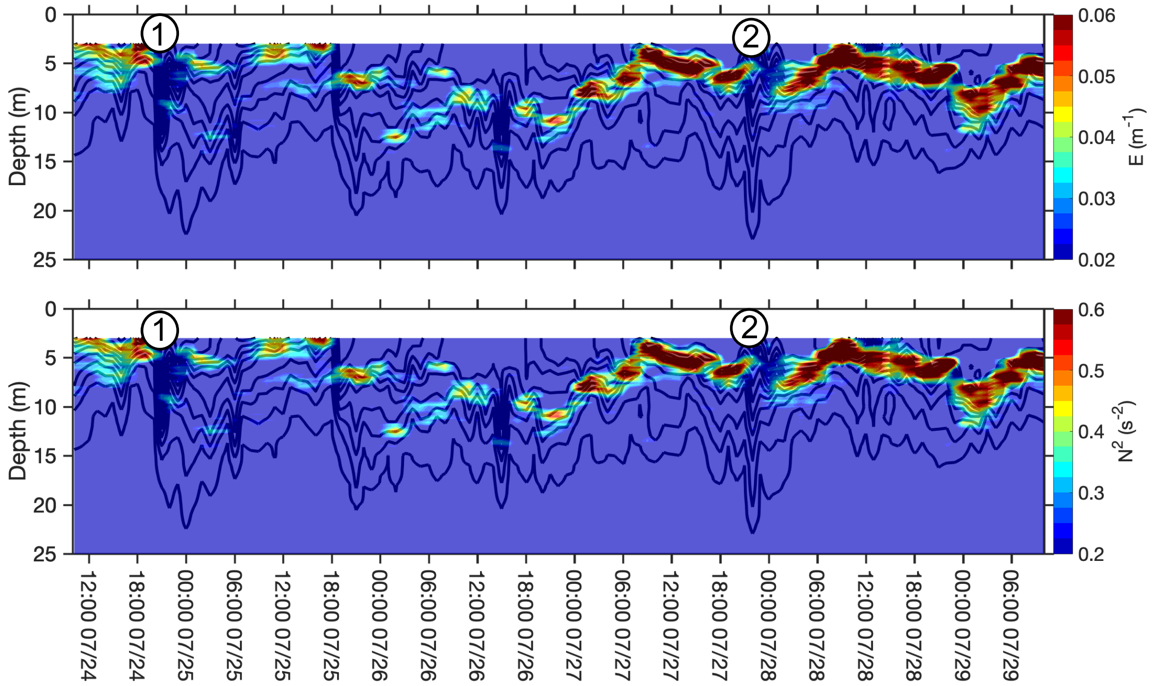

The temporal variability of stratification parameters at ZHJ2. The color contours of the static stability index (upper panel) and buoyancy frequency (lower panel). Salinity contours (black lines) are superimposed. Circled numbers indicate the examples of the NIWs described in the text and in Fig. 5. The buoyancy frequency (N^2) is defined as

$$N^2 = -\frac{g}{\rho_0}\left(\frac{\partial \rho_w}{\partial z}\right)$$

where g is gravitational acceleration, 9.8 m/s^2. ρ_0 is average water density in the measurement, $\partial\rho_w$ and ∂z is the vertical gradient of the water density and the water depth. The z-axis is positive upward.

Supplementary references

1. Saylor, J. H. Studies of Bottom Ekman Layer Processes and Mid-Lake Upwelling in the Laurentian Great Lakes. Water Qual. Res. J. 29, 233–246 (1994)

2. NASA Worldview (https://worldview.earthdata.nasa.gov/)

3. Wang, J., Huang, W., Yang, J., Zhang, H. & Zheng, G. Study of the propagation direction of the internal waves in the South China Sea using satellite images. *Acta Oceanol. Sin.* **32**, 42–50 (2013).
